# Preclinical assessment of oral TLR7 agonist SA-5 in a nonhuman primate model

**DOI:** 10.1172/jci.insight.196809

**Published:** 2025-11-11

**Authors:** Shokichi Takahama, Takahiro Tomiyama, Sachiyo Yoshio, Yuta Nagatsuka, Hirotomo Murakami, Takuto Nogimori, Mami Kochi, Shoko Ochiai, Hidenori Kimura, Akihisa Fukushima, Tatsuya Kanto, Takuya Yamamoto

**Affiliations:** 1 Laboratory of Precision Immunology, Center for Intractable Diseases and ImmunoGenomics, National Institutes of Biomedical Innovation, Health and Nutrition, Osaka, Japan.; 2 Department of Human Immunology and Translational Research, National Institute of Global Health and Medicine, Japan Institute for Health Security, Tokyo, Japan.; 3 Research and Development (Division), Sumitomo Pharma. Co., Ltd., Osaka, Japan.; 4 Department of Liver Diseases, The Research Center for Hepatitis and Immunology, National Institute of Global Health and Medicine, Japan Institute for Health Security, Chiba, Japan.; 5 Laboratory of Aging and Immune Regulation, Graduate School of Pharmaceutical Sciences, and; 6 Department of Virology and Immunology, Graduate School of Medicine, The University of Osaka, Osaka, Japan.; 7 Laboratory of Translational Cancer Immunology and Biology, Next-generation Precision Medicine Research Center, Osaka International Cancer Institute, Osaka, Japan.

**Keywords:** Hepatology, Immunology, Infectious disease, Immunotherapy, Innate immunity

## Abstract

TLR7 agonists are promising immunostimulatory agents for the treatment of chronic infections and cancer. However, their systemic toxicity remains a challenge. In this study, SA-5, a potentially novel liver-targeted, orally available TLR7 agonist, was evaluated for pharmacokinetics, safety, and efficacy in young and aged macaques across 1–10 mg/kg repeated doses. Safety was evaluated through hematologic, biochemical, and flow cytometric profiling, while efficacy was assessed via IFN-α production, gene expression of IFN-stimulated genes, and plasmacytoid dendritic cell activation. A principal component analysis–based (PCA-based) composite scoring system was used to integrate multimodal parameters. SA-5 induced dose-dependent type I IFN with limited systemic inflammation, with 3 mg/kg showing optimal balance. SA-5 had comparable immunostimulatory activity to GS-9620 but with reduced adverse biomarker shifts. In aged macaques, efficacy was maintained with modestly increased safety responses. These findings support SA-5 as a safer next-generation TLR7 agonist effective across age groups, highlighting integrated biomarker profiling in preclinical immunomodulatory drug development.

## Abstract 2

SA-5, a novel oral TLR7 agonist, safely stimulates immune responses in young and aged macaques, supporting its potential as a next-generation immunotherapy.

## Introduction

Pattern recognition receptors (PRRs), including TLRs, play a central role in sensing pathogens and initiating innate immune responses (1, 2). Among the TLRs, TLR7 is localized to intracellular compartments. It recognizes viral single-stranded RNA (ssRNA), leading to the induction of type I IFNs such as IFN-α and IFN-β (3, 4). Given these functions, TLR7 has attracted attention as a potential therapeutic target for chronic viral infections and cancers.

Accordingly, the development of TLR7 agonists has been actively pursued with the aim of inducing type I IFNs. For example, TLR7 ligands have been developed as therapeutic agents for chronic persistent infections such as AIDS and hepatitis B and C. In nonhuman primate models, particularly in the context of HIV-1 infection, TLR7 agonists have been designed to activate latently infected cells. Their use, either as a monotherapy or in combination with neutralizing antibodies, has been reported to exert therapeutic effects (5–7). A Phase Ib clinical trial was conducted in patients receiving combination antiretroviral therapy (cART), in which TLR7 agonists reduced proviral load and delayed viral rebound after treatment interruption. However, it was also suggested that the administration of agonists increased the crosstalk between dendritic cells and NK cells, potentially enhancing cytotoxic activity (5).

Similarly, preclinical nonhuman primate models of hepatitis B demonstrated the potential for a functional cure using TLR7 agonists due to their anti-HBV activity (8–10). However, a human single-dose study of GS-9620 (0.3–12 mg) in healthy volunteers showed systemic type I IFN induction, especially at higher doses (8–12 mg), alongside flu-like symptoms and lymphopenia, highlighting safety concerns (11). Subsequent clinical trials in patients with hepatitis B using lower GS-9620 monotherapy doses (0.3–4 mg, single or multiple) reported mild to moderate adverse events in 58% of participants (12). A phase II trial further indicated systemic type I IFN induction in approximately half of 21 patients, with a similar proportion experiencing flu-like symptoms, reinforcing concerns about the drug’s safety profile (13).

Consequently, the comprehensive evaluation of safety and efficacy is crucial for developing TLR7 agonists into potent immunostimulatory therapeutics. This requires models that faithfully represent human physiology and enable accurate assessment of human TLR7 responses. Nonhuman primates (NHPs) are highly valuable preclinical models for TLR-targeting agent development due to their closer resemblance to human physiology, disease susceptibility, and TLR expression patterns compared with rodents (14). Furthermore, the greater genetic diversity within NHP populations, unlike genetically uniform mouse models, allows for the analysis of interindividual variability in drug responses (15).

In the present study, we utilized cynomolgus monkeys as an NHP model to evaluate the safety and efficacy of SA-5, a potentially novel liver-targeted TLR7 agonist currently in clinical development by our group (16). Similar to GS-9620, SA-5 is an orally administered analog of the pyrimidine derivative DSP-0509 (17–19). SA-5 was specifically designed as a substrate for the organic anion-transporting polypeptides OATP1B1 and OATP1B3, which are expressed on hepatocyte sinusoidal membranes. Following oral administration, the compound travels through the intestinal tract and achieves high liver concentrations via the portal vein (16). Our investigation included assessing the pharmacokinetics (PK) and cytokine-inducing capacity of SA-5 after a single dose. Furthermore, we conducted preclinical studies to evaluate SA-5’s safety and efficacy under repeated dosing. Utilizing time-course samples from these repeated administrations, we performed a comprehensive, integrated analysis of multiple parameters — including hematologic and biochemical tests, ELISA-measured cytokine levels, and immune cell profiling via high-parameter flow cytometry — to create a single-composite index for evaluating both safety and efficacy. Based on this composite index, we also performed a direct comparison with the reference compound GS-9620 and assessed age-dependent effects using aged cynomolgus monkeys.

## Results

### Single and repeated dose-setting study and PK of the oral TLR7 ligand SA-5 in monkeys.

**Figure 1:**
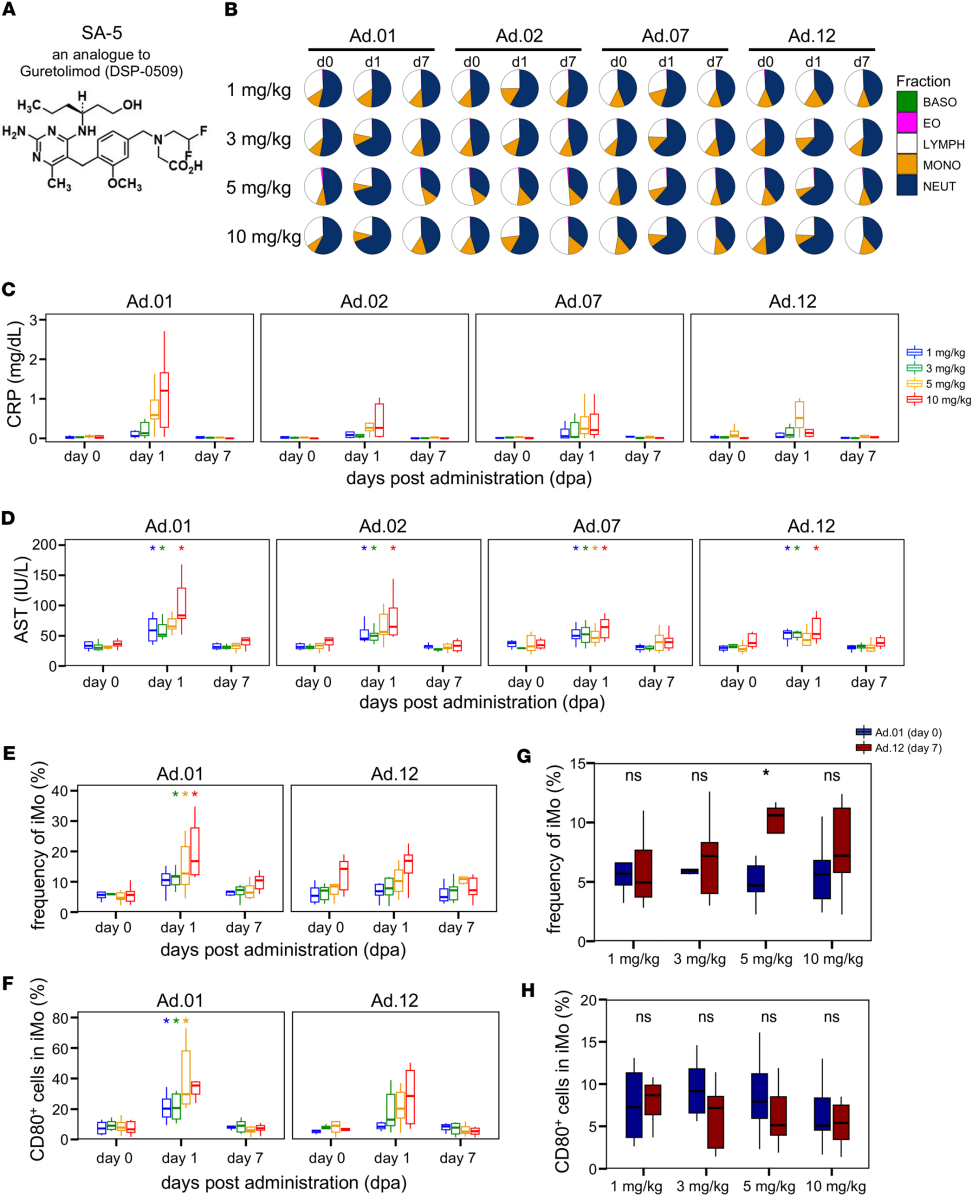
SA-5 safety assessment. Six macaques per dose group were orally administered SA-5 at various dose levels to evaluate the effects of multiple administrations. (**A**) Structural formula of SA-5, an orally active TLR7 agonist structurally related to Guretolimod (DSP-0509), a pyrimidine derivative. (**B**) Pie charts indicate the proportion of main subsets in blood. (**C**) Box plots indicate the changes in blood CRP level (d0, d1, d7) after administration measured by biochemical test. (**D**) Box plots indicate the changes in blood AST level (d0, d1, d7) after administration measured by biochemical test. (**E**) Box plots indicate the changes in the frequency of intermediate monocytes (d0, d1, d7) after administration measured by flow cytometry. (**F**) Box plots indicate the changes in the activation of intermediate monocytes (d0, d1, d7) after administration measured by flow cytometry. (**C**–**F**) Colors indicate doses described in the legend of **B**. Statistical significance relative to day 0 in each administration was determined using paired Wilcoxon tests followed by Benjamini-Hochberg FDR correction across dose groups within each administration and time point; adjusted *q* values are shown. Unless noted otherwise, adjusted *q* values are denoted as: ***q < 0.001, **q < 0.01, *q < 0.05. (**G**) Box plots indicate the changes in the frequency of intermediate monocytes (day 0 at Ad.01 versus day 7 at Ad.12). (**H**) Box plots indicate the changes in the activation of intermediate monocytes (day 0 at Ad.01 versus day 7 at Ad.12). (**G** and **H**) Colors indicate groups as described in the legend of **G**. Statistical significance was determined using the paired Mann-Whitney *U* test comparing Ad.12 day 7 with Ad.01 day 0 (*P < 0.05, **P < 0.01). AST, aspartate aminotransferase; iMo, intermediate monocytes; FCM, flow cytometry; dpa, days postadministration; CBC, complete blood count; CRP, C-reactive protein.

In this study, first, we conducted a single-dose PK study and cytokine analysis of the oral TLR7 ligand SA-5 (Figure 1A) in cynomolgus monkeys (Cohort 1) (Supplemental Figure 1A; supplemental material available online with this article; https://doi.org/10.1172/jci.insight.196809DS1). The plasma concentration of SA-5 increased after its oral administration. In both sexes, the time to reach the maximum plasma concentration (T_max_) was 6–8 hours (h) for all dose groups, except the 300 mg/kg group (Supplemental Figure 2A and Supplemental Table 1). The plasma exposure of SA-5, assessed by area under the plasma concentration-time curve (AUC_0–t_) and maximum plasma concentration (C_max_), increased in a dose-dependent manner (Supplemental Table 1). In all dose groups, a clear induction of type I IFN-α, IL-6, and IP-10 was observed, peaking at 6–8 h after dosing. TNF-α was detectable only in the groups receiving 300 mg/kg or higher, and low levels were detected in the groups receiving 100 mg/kg or lower.

Furthermore, PK and repeated-dose toxicity studies were conducted in cynomolgus monkeys. These animals received once-weekly oral dosing for 4 consecutive weeks, and plasma concentrations were measured at 2, 4, 8, 24, and 48 h after dosing in both the first and fourth weeks (Cohort 2; Supplemental Figure 1A). Consistent with Cohort 1 findings, plasma SA-5 concentrations increased after oral administration. In the 10–100 mg/kg group, T_max_ was 8 h (day 1 [d1] in Supplemental Figure 2C and Supplemental Table 2). Plasma exposure, assessed by AUC_0–t_ and C_max_, showed a dose-dependent increase (Supplemental Table 2 and Supplemental Figure 2C). Notably, no clear correlation was observed between the C_max_ values of the first and fourth doses (Supplemental Figure 2D). Comparison of AUC values indicated that the 10 mg/kg and 30 mg/kg groups were exposed to similar levels of SA-5 (Supplemental Tables 1 and 2). In the 30 mg/kg group, a clear induction of type I IFN was observed immediately after administration, suggesting that a similar response may also occur in the 10 mg/kg group. Furthermore, good tolerability up to 1,000 mg/kg was confirmed with repeated administration for at least 5 weeks. Based on these results, a dosing regimen with a maximum dose of 10 mg/kg was adopted for the subsequent 12-week repeated administration study (Cohort 3; Supplemental Figure 1A).

### Safety profile of the oral TLR7 ligand SA-5.

Subsequently, the safety of repeated SA-5 administration at doses ≤ 10 mg/kg was evaluated (Cohort 3; Supplemental Figure 1A). Six cynomolgus monkeys received oral SA-5 (1, 3, 5, or 10 mg/kg) every other week for 12 weeks, with blood sampling before dosing, 1 day (d) after dosing, and 7 d after dosing (prior to the next administration); plasma and peripheral blood mononuclear cells (PBMCs) were isolated for subsequent analyses. To reduce sampling burden, only drug administration occurred at weeks 5, 6, 9, and 10.

Throughout the study, no notable changes in body weight were observed (Supplemental Figure 3). Hematologic parameters were monitored after each SA-5 dose to evaluate drug-related changes (Figure 1B and Supplemental Figure 4), using statistical analyses comparing postdosing values to both the first dosing day (Ad.0_d0) and the respective dosing day for each administration. The first administration showed a marked decrease in peripheral blood lymphocyte counts at ≥ 3 mg/kg 1 d after dosing (Supplemental Figure 4). However, from the second administration onward, no significant changes were detected at ≤ 3 mg/kg. At 5 and 10 mg/kg, considerable decreases were observed after the twelfth and seventh administrations, respectively, but resolved within 1 week. Leukocyte subset analysis indicated a trend toward decreased lymphocyte and increased neutrophil fractions 1 d after administration without consistent statistical significance across dose groups (Figure 1B and Supplemental Figure 4B). Other hematologic parameters showed no notable adverse effects (Supplemental Figure 4, C and D).

Biochemical inflammatory markers, analyzed similarly, revealed a dose-dependent elevation in C-reactive protein (CRP), with trend-level transient increases 1 d after administration across all dose groups (*q* = 0.059–0.083), returning to baseline within 1 week (Figure 1C). Liver injury markers (aspartate aminotransferase [AST] and lactate dehydrogenase [LDH]) were markedly elevated 1 d after administration across all dose groups (1–10 mg/kg) but returned to predosing levels by 1 week (Figure 1D and Supplemental Figure 5A). Comparisons between the first administration and values 7 d after subsequent administrations showed no significant differences, suggesting no irreversible effects (Supplemental Figure 5A). A positive correlation between AST and LDH and a partial correlation between AST and CRP were observed at doses ≥ 3 mg/kg 1 d after administration (Supplemental Figure 5B). While other parameters showed transient elevations, these were within expected inflammatory response ranges (Supplemental Figure 5, C and D). Minor, transient deviations in some liver function and inflammatory markers were noted immediately after dosing but subsided and returned to normal. Other biochemical parameters showed no notable adverse effects (Supplemental Figure 5, C and D).

Plasma levels of proinflammatory cytokines IL-6 and TNF showed induction in some high-dose individuals (5 and 10 mg/kg) 1 d after administration but no statistically significant changes across dose groups (Supplemental Figure 6A). Similarly, PBMC IL-6 and TNF expression by reverse transcription PCR showed no clear dose-dependent increase (Supplemental Figure 6B), indicating no systemic inflammatory cytokine induction up to 10 mg/kg.

High-parameter flow cytometry of PBMCs assessed SA-5’s effect on cell frequency and phenotype, focusing on monocytes. Intermediate monocytes (iMo; CD14^+^CD16^+^), associated with inflammation and antigen presentation, showed a transient increase 1 d after dosing, returning to baseline by d7 (Figure 1E and Supplemental Figure 7, A and B). Notable iMo increases occurred at ≥ 3 mg/kg after the first dose (Figure 1E). Activation markers CD80, CCR7, and CD169/Siglec-1 on iMos increased 1 d after dosing and returned to baseline by d7 (Figure 1F and Supplemental Figure 7D). Classical and nonclassical monocyte subsets were also analyzed (Supplemental Figure 7). While frequency changes were most prominent and consistent in iMo, dose-dependent increases in activation markers at d1 were detectable across all 3 subsets. This indicates that iMo provide the most sensitive marker of SA-5–induced myeloid activation, as they combine both expansion in frequency and upregulation of activation markers. Upon comparing the initial and final time points, a marked iMo increase was observed in the 5 mg/kg group, but no changes in activation marker expression were noted at any dose (Figure 1, G and H).

Collectively, the transient changes in monocyte composition and iMo activation following SA-5 administration suggest a transient inflammatory response, consistent with hematologic and biochemical findings. Importantly, no statistically significant differences were observed between the initial and final dosing time points for any evaluated parameter, indicating that the observed safety-related changes during the 12 repeated administrations were transient.

### Evaluation of safety-related biomarkers.

**Figure 2:**
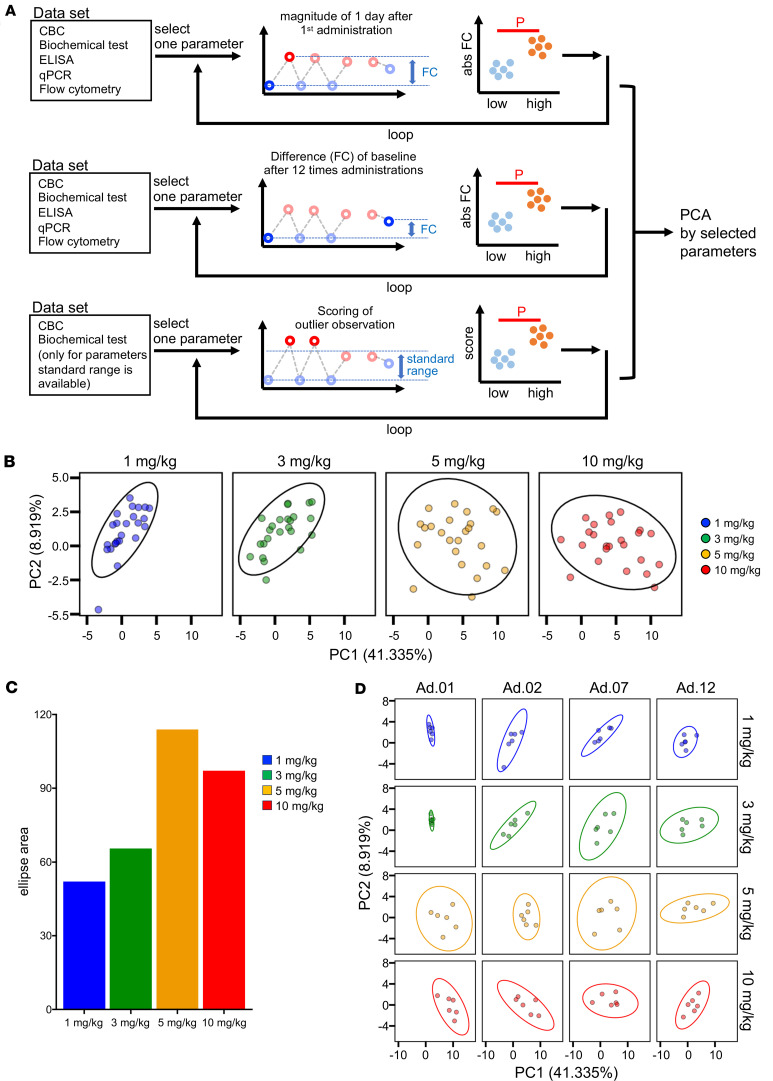
Multiparameter safety assessment of multiple SA-5 administration of different doses in cynomolgus macaque. (**A**) Schematic depiction of the analysis pipeline. (**B**) Principal component analysis (PCA) plot generated using values of selected parameters based on the statistically significant data shown in Supplemental Figure 9. Probability ellipses indicate the area where 90% of a distribution lies. The colors indicate the dose. (**C**) Bar plots indicate the relative size of the ellipse area shown in **B**. Colors indicate each dose. (**D**) PCA plot generated using values of selected parameters based on the statistically significant data. Colors indicate each dose as in **B**. PCA, principal component analysis; PC1, principal component 1.

Next, to identify potential safety biomarkers, we integrated data from various analyses, comprehensively evaluating drug-induced responses, repeated dosing effects on baseline levels, and deviations from reference values by performing a group-wise comparison between low-dose (1–3 mg/kg) and high-dose (5–10 mg/kg) groups to extract parameters exhibiting dose-dependent changes (Figure 2A). Parameter extraction relied on 3 indices: (a) fold change (FC) between predosing and 1 d after first administration, (b) FC between predosing and 7 d after the twelfth and final administration, and (c) a deviation score reflecting the extent of deviation from the established reference values (Supplemental Figure 8).

For hematologic and biochemical parameters, a deviation scoring system was applied to 35 parameters with established reference ranges. The range unit was defined as the midpoint between the upper and lower reference limits. Deviations were categorized and scored: minor deviation (within the reference range limits = score 1), moderate deviation (exceeding the reference range by ≥ 1 range unit = score 2), and marked deviation (exceeding the reference range by ≥ 2 range units = score 4), with separate scores for deviations above and below the range (Supplemental Figure 8). Calculating the difference in deviation scores (delta scores) revealed a trend toward a dose-dependent increase (Supplemental Figure 8, B and C).

Out of 132 parameters, 27 were identified as reflecting dose-responsive changes: 20 were associated with responses after the first administration, 3 reflected fold changes after the final administration, and 4 were derived from the deviation score index (Supplemental Figure 9). For these selected parameters, we extracted: (a) FC between predose and 1 d after dose for each administration, (b) FC relative to the baseline value prior to the first administration across all dosing points, and (c) deviation scores from reference values. These data were subjected to principal component analysis (PCA) to generate datasets for each individual at each dosing point. PCA plotting of post-dosing d1 values for each administration (*n* = 6 animals × 4 dosing points = 24 data points) showed a dose-dependent expansion in distribution (circle size), with PC1 shifting positively and PC2 shifting negatively with increasing dose (Figure 2B). Based on probabilistic ellipse areas, a dose-dependent expansion in distribution was observed (Figure 2C), suggesting increasing interindividual variability in off-target responses with dose, plateauing at 5 mg/kg. To assess repeated dosing effects, PCA plots were generated for the first, second, seventh, and twelfth administrations. In animals receiving ≥ 5 mg/kg, the 90% probabilistic ellipses after the first and second doses were markedly shifted negatively, indicating the potential induction of qualitatively distinct off-target responses compared with ≤ 3 mg/kg (Figure 2D).

### Evaluation of the efficacy profile of SA-5, an oral TLR7 agonist.

**Figure 3:**
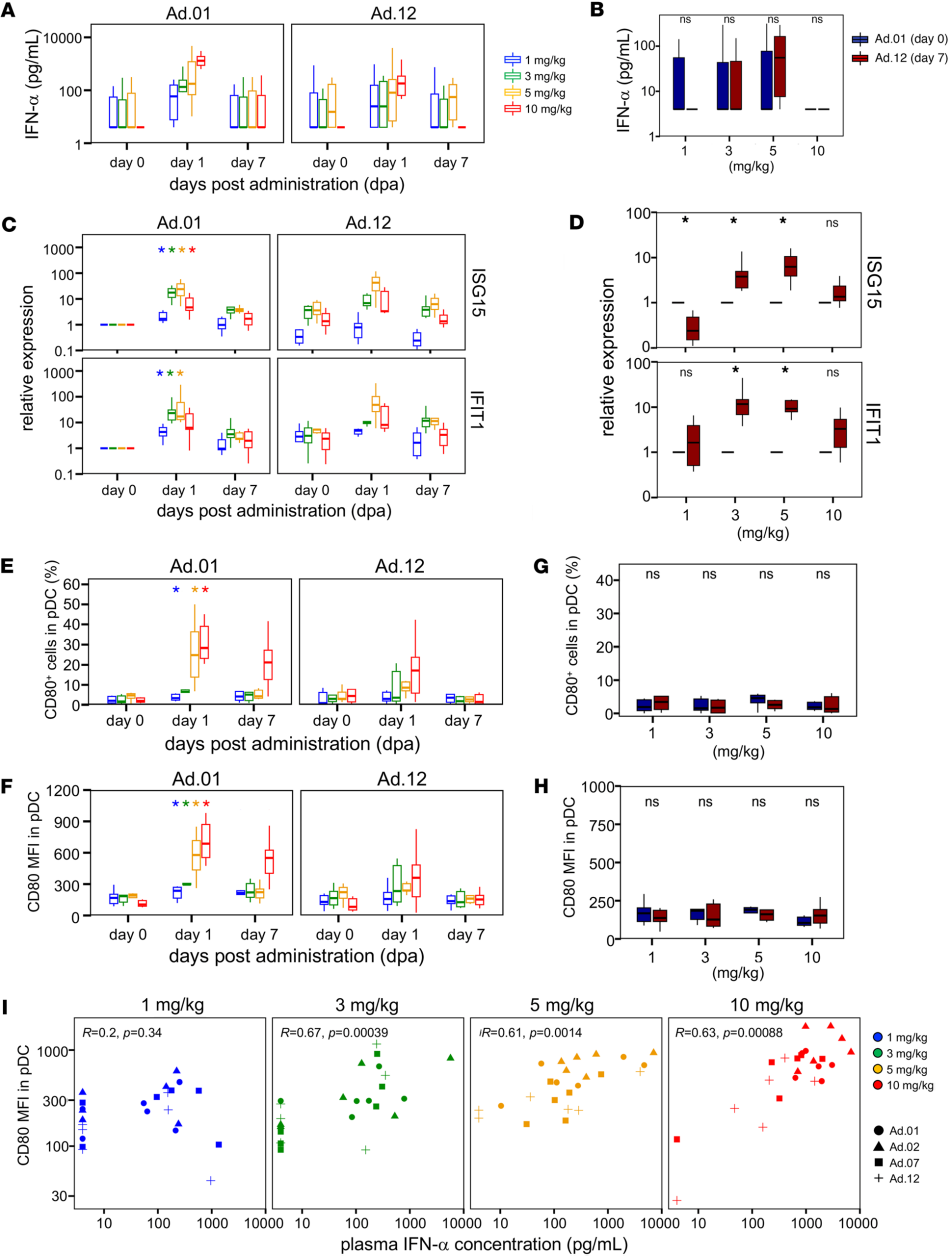
Efficacy assessment of multiple SA-5 administration of different doses in cynomolgus macaque. Macaques (*n* = per 6 dose) received oral SA-5. Box plots show: (**A**) plasma IFN-α level (d0, d1, d7) measured by ELISA; (**B**) IFN-α levels at Ad.01 day 0 versus Ad.12 day 7; (**C**) representative IFN-induced gene expression (days 0, 1, and 7) measured by qRT-PCR; (**D**) gene expression at Ad.01 day 0 versus Ad.12 day 7; (**E**) DC activation (CD80^+^ frequency) (days 0, 1, and 7) as measured by flow cytometry; (**F**) DC activation (CD80 MFI in pDC) (days 0, 1, and 7); (**G**) CD80^+^ frequency at Ad.01 day 0 versus Ad.12 day 7; and (**H**) CD80 MFI in pDC at Ad.01 day 0 versus Ad.12 day 7. (**I**) Correlation between IFN-α and CD80 MFI in pDC at 1 dpa, including data from all administrations (Ad.01–Ad.12), thereby increasing statistical power. (**A**, **C**, **E**, and **F**) Colors denote dose groups. Statistical significance relative to day 0 in each administration was determined using paired Wilcoxon tests followed by Benjamini–Hochberg FDR correction across dose groups within each administration and time point; adjusted *q*-values are shown. Unless noted otherwise, adjusted *q*-values are denoted as: *** *q* < 0.001, ** *q* < 0.01, * *q* < 0.05. (**B**, **D**, **G**, and **H**) Statistical significance was determined by paired Mann-Whitney *U* test comparing Ad.12 day 7 with Ad.01 day 0 (*P < 0.05, **P < 0.01; ns, not significant). (**I**) Correlation was evaluated using the Spearman’s rank correlation test. Colors indicate each dose as described in **A**. IFN-α, IFN-alpha; qRT-PCR, quantitative reverse transcription PCR; pDC, plasmacytoid dendritic cell; MFI, median fluorescence intensity; ISG, IFN-stimulated gene; IFIT1, IFN-induced protein with tetratricopeptide repeats 1.

Next, we investigated the induction of plasma type I IFN production, a key efficacy-related outcome of SA-5, resulting from its target TLR7 activation. ELISA measurement of plasma IFN-α levels after SA-5 administration showed a consistent increase 1 d after dosing across all time points, exhibiting a clear dose-dependent trend (Figure 3A and Supplemental Figure 10A). At 1 and 3 mg/kg, 1 monkey in each group showed no detectable IFN response, corresponding to no statistical significance at 1 mg/kg and a trend at 3 mg/kg (*q* = 0.079). Conversely, at 5 and 10 mg/kg, all animals displayed a robust IFN-α response after the first and second administrations, showing trend-level increases (*q* = 0.063) after multiple-comparison correction. However, repeated dosing led to a gradual attenuation of this response over time. Furthermore, comparing baseline and 7 d after final administration revealed no statistically significant differences at any dose (Figure 3B).

To further support these findings, qPCR in PBMCs examined type I IFN and IFN-stimulated gene (ISG) expression. Significant upregulation of ISGs IFIT1 and ISG15 occurred 1 d after first administration across all dose groups (Figure 3C and Supplemental Figure 10B). Notably, in the 5 mg/kg group, this upregulation was consistently observed 1 d after each administration (Supplemental Figure 10B). While the 5 mg/kg group had the strongest gene induction after both the first and twelfth administrations, the 10 mg/kg group showed weaker ISG upregulation than the 5 mg/kg group 1 d after the initial dose, suggesting potential suppression at excessive dosing. Comparing gene expression between the first administration and 7 d after final administration showed a marked increase at 3 and 5 mg/kg (Figure 3D).

Furthermore, we evaluated the abundance and activation of plasmacytoid dendritic cells (pDCs), which are known to highly express TLR7, a target of SA-5, major type I IFN sources, using high-parameter flow cytometry. A marked increase in pDC frequency and activation marker CD80 expression was observed 1 d after first administration at all doses, with a clear dose-dependent magnitude (Figure 3, E and F, and Supplemental Figure 10D). pDC activation decreased with repeated 5 and 10 mg/kg doses. After the twelfth dose, considerable activation (MFI) was only observed at 10 mg/kg (Supplemental Figure 10E), while frequency-based significance was only noted at 3 mg/kg. Myeloid DCs (mDCs) showed similar activation patterns to pDCs, with no significant activation at 1 mg/kg after the first dose but clear activation at ≥ 3 mg/kg.

Comparing the first administration and 7 d after final administration revealed no statistically significant changes in pDCs or mDCs at any dose (Figure 3, G and H). Analyzing the relationship between plasma IFN-α and ISG expression 1 d after administration showed no significant correlation for *IFIT1* or *ISG15* (Supplemental Figure 10C), suggesting their potential as independent biomarkers of type I IFN pathway activation. In contrast, a strong positive correlation was observed between plasma IFN-α and pDC activation at doses ≥ 3 mg/kg (Figure 3I).

### Evaluation of efficacy-related biomarkers.

**Figure 4:**
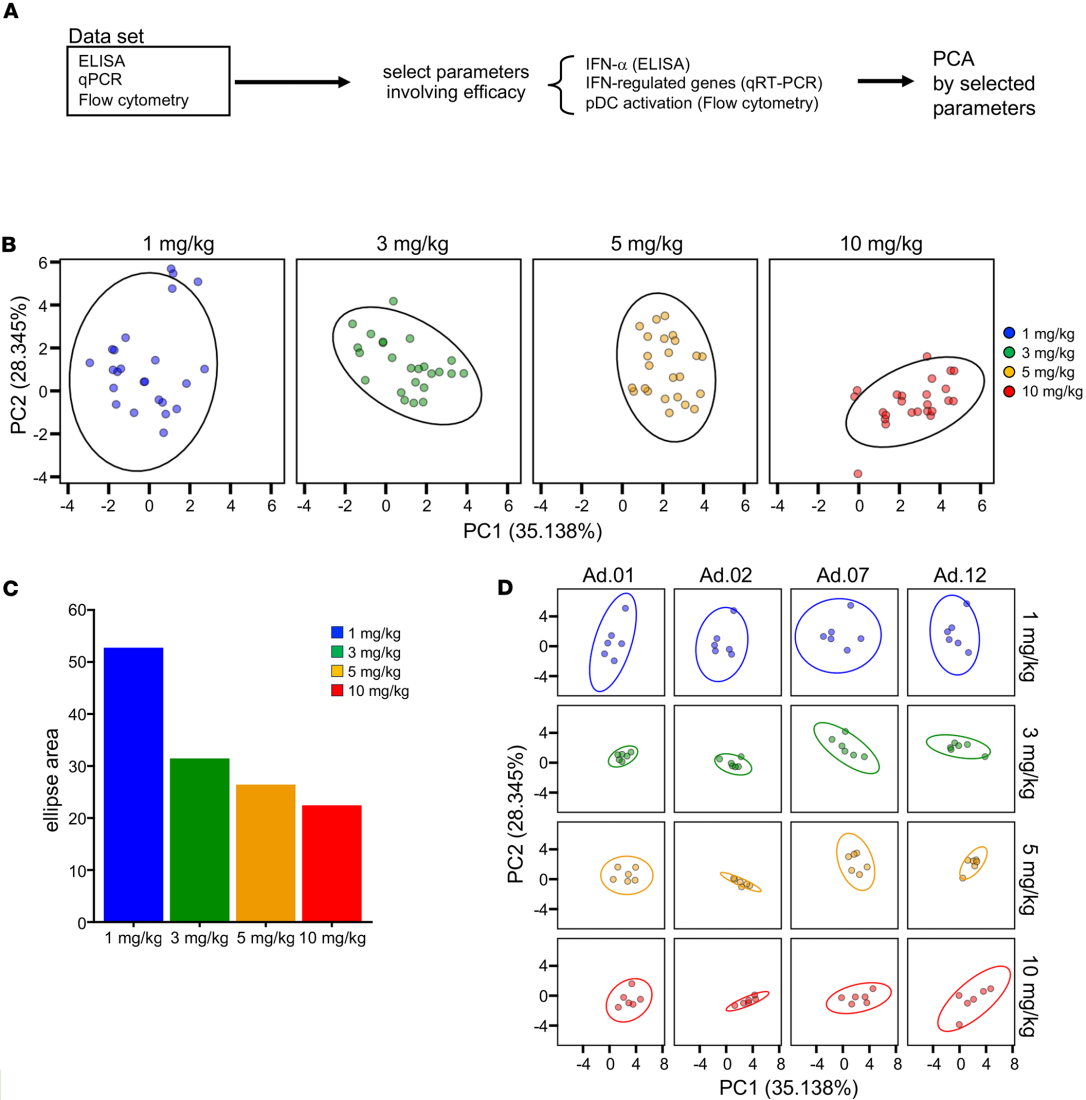
Multiparameter efficacy assessment of multiple SA-5 administration of different doses in cynomolgus macaque. (**A**) Schematic depiction of the analysis pipeline. (**B**) Principal component analysis (PCA) plot generated using values of selected parameters based on the statistically significant data shown in Figure 2C. Probability ellipses indicate the area where 90% of a distribution lies. The colors indicate the dose. (**C**) Bar plots indicate the relative size of the ellipse area shown in **D**. Colors indicate each dose as in **B**. (**D**) PCA plot generated using values of selected parameters based on the statistically significant data shown in Figure 2C. The colors indicate the dose. PCA, principal component analysis; ISG, IFN-stimulated gene.

Finally, to identify efficacy-related biomarkers, we integrated data from various analyses, selecting 9 markers associated with type I IFN-α induction: plasma IFN-α levels, expression of 7 ISGs in PBMCs via qPCR, and the frequency of activation markers on pDCs, the primary type I IFN source. These markers underwent PCA for comprehensive evaluation (Figure 4A). The PCA revealed a dose-dependent shift, with PC1 increasing positively and PC2 shifting negatively (Figure 4B). The 90% probabilistic ellipse area decreased dose-dependently, indicating convergence toward a distinct phenotypic pattern at higher doses (Figure 4C). Separate PCA plots for the first, second, seventh, and twelfth administrations showed 90% ellipse convergence after the first and second doses at doses ≥ 3 mg/kg, while no such convergence occurred at 1 mg/kg across all time points. This suggests that efficacy-related immune activation occurred at doses of 3 mg/kg or higher (Figure 4D). Notably, the degree of convergence decreased after the seventh and twelfth administrations. Collectively, these findings indicate that SA-5 has the potential to activate the type I IFN pathway when administered at ≥ 3 mg/kg for at least 2 doses.

### Integrated analysis of safety and efficacy.

**Figure 5:**
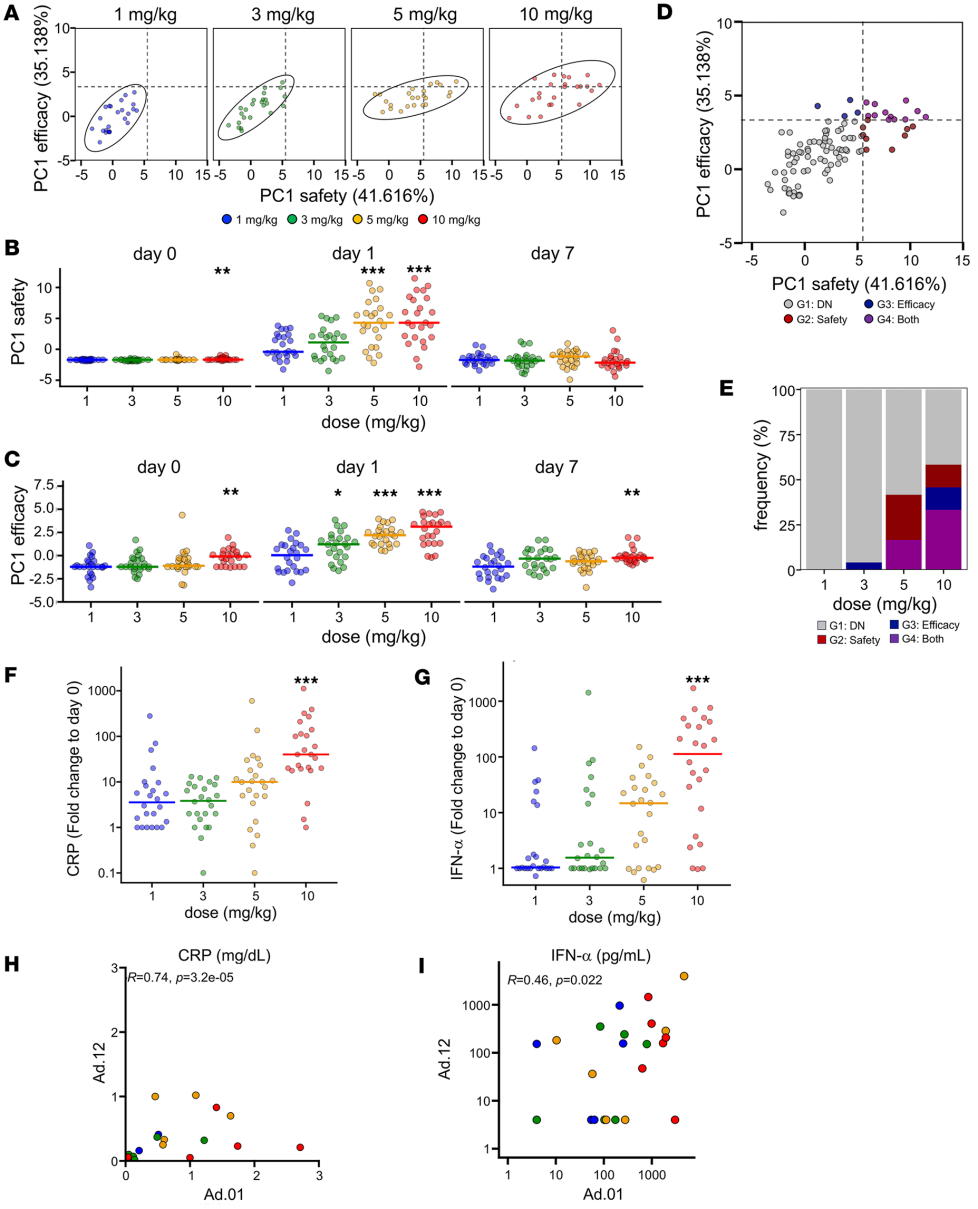
Integrated analysis of safety and efficacy. (**A**) Scatter plot indicates PC1 values from 2 principal component analyses (PCAs) for safety and efficacy. Probability ellipses indicate areas encompassing 90% of the distribution. Dashed lines indicate the boundaries of 4 regions defined by the ellipse border at 1 mg/kg. Colors indicate each dose. (**B**) PC1 safety values in each dose group. (**C**) PC1 efficacy values in each dose group. (**D**) Scatter indicates PC1 values for safety and efficacy, as in **A**, but not separated by dose. Colors indicate each region. (**E**) Bar plots indicate the frequency of macaques in each region as in **D**. (**F**) CRP scores in each dose group. (**G**) IFN-α scores in each dose group. (**H**) Scatter plot indicates the correlation between CRP values at Ad.01 and Ad.12. (**I**) Scatter plot indicates the correlation between IFN-α values at Ad.01 and Ad.12. (**B** and **C**) Statistical significance among dose groups was determined using the Wilcoxon rank-sum test stratified by administration (van Elteren), comparing each dose group with the 1 mg/kg group. (**F** and **G**) Statistical significance among dose groups based on fold-change values at day 1 (relative to day 0) was determined using the same test. *P* values were adjusted for multiple comparisons among dose groups using the Benjamini-Hochberg FDR correction; adjusted *q*values are shown. Unless noted otherwise, adjusted *q*values are denoted as: ***q < 0.001, **q < 0.01, *q < 0.05. (**H** and **I**) Statistical significance was determined using the Spearman’s rank correlation test. (**A**–**C**, **F**, and **G**) Colors indicate each dose as in **A**. (**D** and **E**) Colors indicate each region as in **D**. PCA, principal component analysis; PC1, principal component 1; CRP, C-reactive protein.

To evaluate the relationship between safety and efficacy in individual cynomolgus monkeys, we plotted the PC1 values obtained from the respective PCAs of safety (PC1 safety) and efficacy (PC1 efficacy) parameters (Figure 5A). This visualization revealed a dose-dependent upward-sloping distribution pattern.

Using the values at 1 mg/kg as a reference, statistical comparisons were performed for each PC1 axis. A significant increase in PC1 safety was observed 1 d after administration at doses of 5 and 10 mg/kg, indicating that safety was likely maintained at doses of up to 3 mg/kg (Figure 5B). In contrast, for efficacy, considerable differences were observed at doses of 3 mg/kg and above (Figure 5C).

To further assess the magnitude of individual responses in cynomolgus monkeys, we classified the animals into 4 groups based on PC1 safety and PC1 efficacy values, using the maximum value of the 1 mg/kg probabilistic ellipse as a threshold. The groups were defined as follows: low safety and low efficacy (G1: DN); high safety and low efficacy (G2: Safety); low safety and high efficacy (G3: Efficacy); and high safety and efficacy (G4: Both) (Figure 5D). This classification revealed that animals exhibiting high efficacy (PC1 efficacy) began to appear at a dose of 3 mg/kg, whereas animals showing elevated safety responses (PC1 safety) were evident from 5 mg/kg onward (Figure 5E).

To validate the PCA results, we examined the dose dependency of representative parameters for safety and efficacy: CRP and IFN-α, respectively. Both markers showed a dose-dependent trend toward increased median values (Figure 5, F and G). Furthermore, correlation analyses between the first and twelfth administrations revealed a strong positive correlation for both CRP and IFN-α (Figure 5, H and I), suggesting that the integrated analysis captured consistent trends across repeated dosing.

Based on these findings, a dose of 3 mg/kg was concluded to represent the optimal balance between safety and efficacy, as it demonstrated therapeutic activity while maintaining an acceptable safety profile.

### Comparative evaluation of the predecessor TLR7 ligand GS-9620 and SA-5.

**Figure 6:**
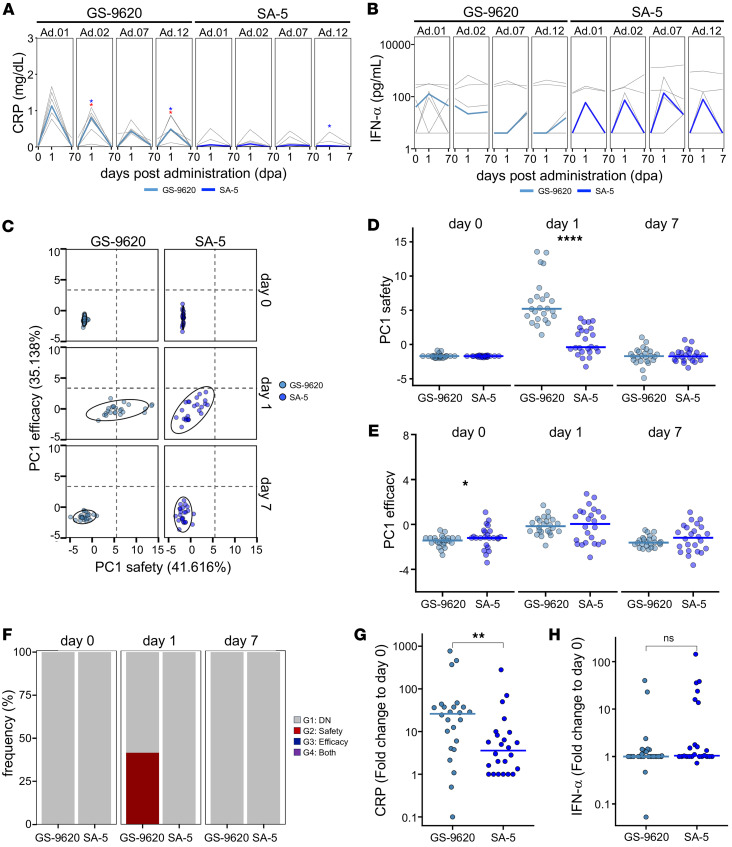
Direct comparison of SA-5 versus GS-9620. (**A**) Line plots indicate changes in blood CRP levels (d0, d1, d7) after administration, measured by biochemical testing. (**B**) Line plots indicate changes in plasma IFN-α levels (d0, d1, d7) after administration, measured by ELISA. (**C**) Scatter plot indicates PC1 values from 2 principal component analyses (PCAs) for safety and efficacy. Probability ellipses indicate areas encompassing 90% of the distribution. Dashed lines indicate the borders of the 4 regions defined in Figure 5. Colors indicate the adjuvant. (**D**) PC1safety values in each adjuvant group. (**E**) PC1efficacy values in each adjuvant group. (**F**) Bar plots indicate the frequency of macaques in each region as in Figure 5E. (**G**) CRP scores in each adjuvant group. (**H**) IFN-α scores in each adjuvant group. (**A** and **B**) Statistical significance was determined using the paired Mann-Whitney *U* test for comparisons with d0. Blue asterisks indicate comparisons with day 0 of Administration 1 (Ad.01), and red asterisks indicate comparisons with day 0 of each corresponding administration. (*P < 0.05). (**D**, **E**, **G**, and **H**) Statistical significance was determined using the Mann-Whitney *U* test for comparisons between GS-9620 and SA-5 at each corresponding time point (*P < 0.05, **P < 0.01, ****P < 0.0001). (**C**–**E**, **G**, and **H**) Colors indicate each adjuvant as in **C**. (**F**) Colors indicate each region as in Figure 5E. PCA, principal component analysis; CRP, C-reactive protein; PC1, principal component 1.

Next, a comparative analysis was conducted using the existing agent GS-9620. Body weights remained stable throughout the study period in both treatment groups (Supplemental Figure 11, A and B). Evaluation of serum CRP levels as a safety marker revealed no significant changes in the SA-5 group at 1 mg/kg. In contrast, the GS-9620 group exhibited remarkable increases at multiple time points (Figure 6A). In contrast, regarding IFN-α as an efficacy marker, the SA-5 group showed a trend toward induction 1 d after dosing, while the GS-9620 group demonstrated no clear induction (“blip”) (Figure 6B).

Using the same PCA-based approach shown in Figure 5, we plotted the safety and efficacy responses for each group. Both groups exhibited no shifts 1 d after dosing; however, while safety scores in the SA-5 group remained below the defined threshold, those in the GS-9620 group markedly exceeded this threshold (Figure 6C). Group-wise statistical comparisons revealed no significant difference in PC1 efficacy between the 2 groups. In contrast, PC1 safety was markedly elevated in the GS-9620 group 1 d after dosing, suggesting that SA-5 had a considerably better safety profile than GS-9620 (Figure 6, D and E). Furthermore, using the classification method described in Figure 5E, safety-efficacy profiling showed that approximately half of the animals in the GS-9620 group fell into categories associated with safety concerns (Figure 6F).

To validate the PCA results, representative safety and efficacy parameters — CRP and IFN-α — were compared between the 2 groups. While no significant difference in IFN-α levels was observed, serum CRP levels were notably higher in the GS-9620 group compared with the SA-5 group (Figure 6, G and H). Additionally, the deviation score was markedly higher in the GS-9620 group (Supplemental Figure 11C). Collectively, a comprehensive comparison at an equivalent dose level suggested a superior safety profile for SA-5.

### Investigation in aged NHPs.

**Figure 7:**
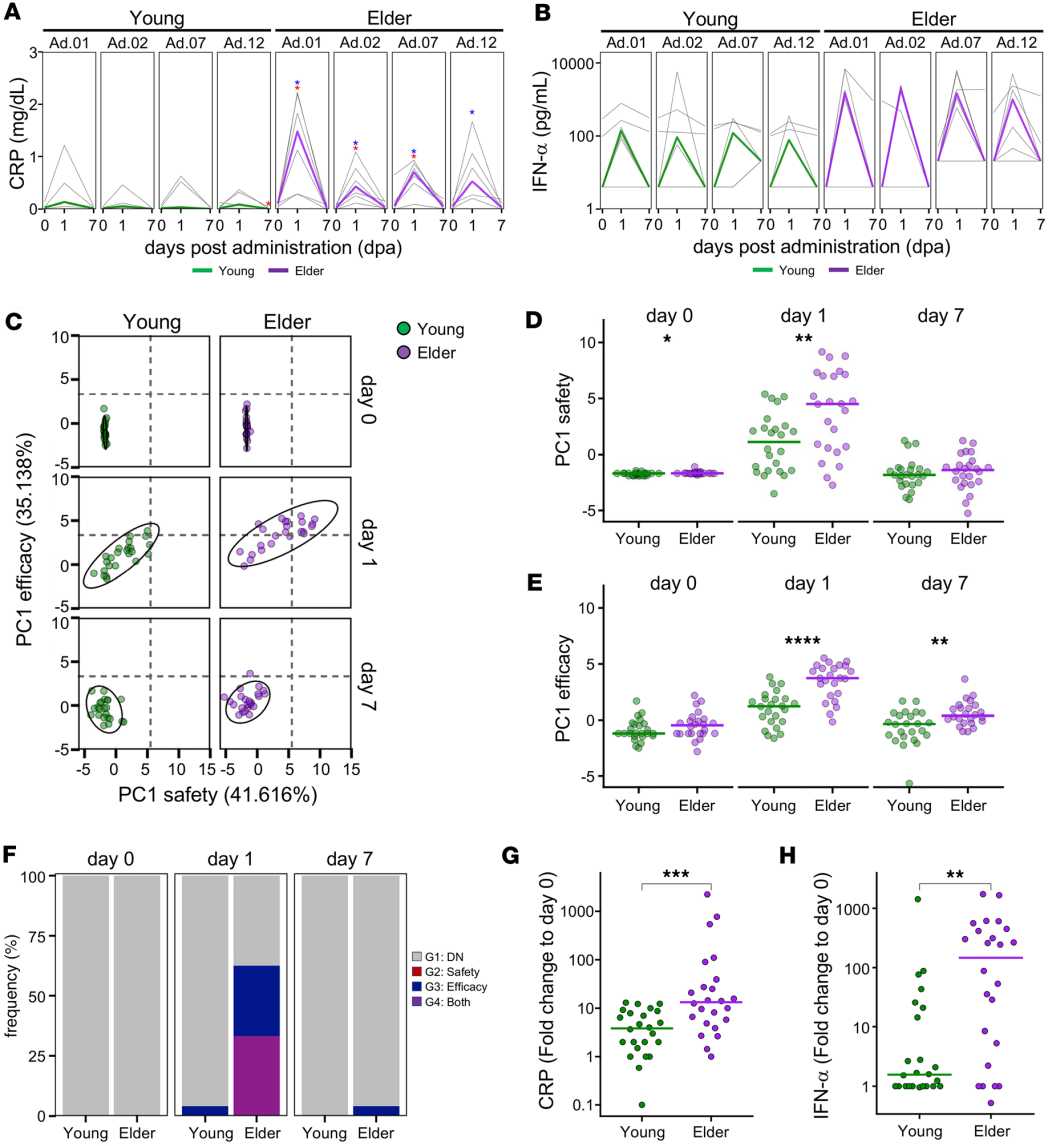
Direct comparison of young versus older populations. (**A**) Line plots indicate changes in blood CRP levels (d0, d1, d7) after administration, measured by biochemical testing. (**B**) Line plots indicate changes in plasma IFN-α levels (d0, d1, d7) after administration, measured by ELISA. (**C**) Scatter plot indicates PC1 values from 2 principal component analyses (PCAs) for safety and efficacy. Probability ellipses indicate areas encompassing 90% of the distribution. Dashed lines indicate the borders of the 4 regions defined in Figure 5. (**D**) PC1 safety values in each age group. (**E**) PC1 efficacy values in each age group. (**F**) Bar plots indicate the frequency of macaques in each region as in Figure 5E. (**G**) CRP scores in each age group. (**H**) IFN-α scores in each age group. (**A** and **B**) Statistical significance was determined using the paired Mann-Whitney *U* test for comparisons with d0. Blue asterisks indicate comparisons with d0 of Administration 1 (Ad.01), and red asterisks indicate comparisons with d0 of each corresponding administration (*P < 0.05). (**D**, **E**, **G**, and **H**) Statistical significance was determined using the Mann-Whitney *U* test for comparisons between Young and Elder at each corresponding time point (*P < 0.05, **P < 0.01, ***P < 0.001, ****P < 0.0001). (**A**–**E**, **G**, and **H**) Colors indicate each age group (young versus older) as in **C**. (**F**) Colors indicate each region as in Figure 5E. CRP, C-reactive protein; PCA, principal component analysis; PC1, principal component 1.

Finally, we conducted a comparative analysis at the 3 mg/kg dose — identified as both safe and effective — to evaluate age-related differences, focusing on an aged group (≥ 20 years old; Supplemental Figure 12A). Except for 1 animal in the aged group, the body weight remained stable throughout the study period in both the young and aged groups (Supplemental Figure 12, B and C). The evaluation of serum CRP levels as a safety marker revealed no significant changes in the young group. In contrast, the aged group exhibited remarkable increases at multiple time points (Figure 7A). Additionally, the deviation score was considerably higher in the aged group (Supplemental Figure 12D).

In contrast, for the efficacy marker IFN-α, both young and aged groups exhibited a trend toward induction 1 d after dosing (Figure 7B). Using the PCA-based analysis described in Figure 5, we plotted the safety and efficacy responses, which revealed clear shifts 1 d after administration in both groups. Notably, the aged group exhibited a positive directional shift along both axes (Figure 7C). Group-wise statistical comparisons showed a significant increase in the aged group 1 d after dosing, suggesting a modest increase in safety-related concerns. Simultaneously, efficacy was maintained (Figure 7, D and E). When the safety and efficacy classification was performed using the framework described in Figure 5E, none of the individuals in either group fell into the category associated with safety concerns alone. Furthermore, approximately half of the animals in the aged group showed an efficacy profile (Figure 7F).

To validate the PCA results, representative safety and efficacy parameters — CRP and IFN-α — were compared between the young and aged groups. Both these parameters were markedly higher in the aged group (Figure 7, G and H). Collectively, these findings from a comprehensive comparison at the same dose level suggest that, although safety-related concerns may increase, SA-5 retains the potential to elicit efficacy even in aged individuals.

## Discussion

TLR7 agonists are actively being developed as therapeutic agents for chronic persistent infections and cancer (20–23). In particular, their potential as treatments for chronic infections such as HBV and HIV, either as monotherapy or in combination with other therapeutic strategies, has been explored, and some candidates have already advanced to clinical trials in humans (5, 9, 12, 13, 24–26). However, to date, no TLR7 agonist has received regulatory approval for any indication, primarily due to safety or efficacy limitations. Therefore, the development of novel agents that can overcome the shortcomings of the existing compounds is critical.

In this study, we conducted preclinical evaluations of SA-5 in cynomolgus monkeys. PK profiling of SA-5 was performed, and a comprehensive safety assessment was conducted by selecting dose-dependent parameters and applying an integrated analysis of multilayered datasets. Across the tested doses of 1, 3, 5, and 10 mg/kg, transient reductions in peripheral blood lymphocytes and increases in biochemical markers and inflammatory response indicators were observed, with most parameters returned to baseline within 1 week of administration. Among monocyte subsets, iMo, which are involved in inflammatory cytokine production, showed the most pronounced and consistent changes. They exhibited dose-dependent increases in frequency and activation markers at d1, with values generally resolving by d7, except for a sustained elevation at 10 mg/kg. Classical and nonclassical monocytes also displayed activation at d1, albeit less prominently (Supplemental Figure 7). Therefore, iMo represent the most sensitive readout of SA-5–induced myeloid activation. To assess safety from multiple perspectives, including short- and long-term effects, as well as deviations from reference values, we evaluated changes from baseline after 12 repeated doses, dose-by-dose fluctuations before and after administration, and deviation scores. PCA revealed a dose-dependent expansion of the probabilistic ellipses. Notably, after the first and second administrations, animals receiving ≥ 5 mg/kg showed distributions that were markedly different from those in the 3 mg/kg group, suggesting the possibility of qualitatively distinct off-target responses at higher doses.

In preliminary single-dose no-observed-adverse-effect level (NOAEL) studies, administration of SA-5 up to 1,000 mg/kg appeared tolerable, although the very small cohort size (*n* = 2 per group; Supplemental Figures 1 and 2) precluded robust statistical conclusions. On this basis, we set 10 mg/kg as the upper limit for the repeated-dose evaluation, as it was the lowest dose at which safety-related changes were initially noted in the PK study. In a 12-week repeated-dose study with larger cohorts (*n* = 6/group, 1–10 mg/kg), safety deviations — including CRP elevation (Figure 1C and Supplemental Figure 5A), dose-dependent expansion of the safety PCA distribution (Figure 2, B–D), and iMo alterations (Figure 1, F and G, and Supplemental Figure 7) — emerged at ≥ 5 mg/kg, whereas clear efficacy, evidenced by IFN-α induction and pDC activation (Figure 3I), was already detectable at 3 mg/kg. Accordingly, integrated analysis demonstrated that 3 mg/kg represented the optimal balance between safety and efficacy. Importantly, in aged macaques, CRP elevations were observed even at 3 mg/kg, highlighting the need for careful monitoring in older populations. While these findings led us to select 3 mg/kg as the recommended dose for further pursuit, the more favorable safety profile of SA-5 compared with GS-9620 suggests that exploration of higher doses (>5 mg/kg) may still be feasible in future studies.

Extensive preclinical and clinical data have been accumulated on GS-9620, a previously developed TLR7 agonist. In human studies, GS-9620 was administered at doses ranging from 1 to 12 mg in healthy individuals, as well as in patients with HBV or HIV infection. Most adverse events were classified as grade 1 or 2, and the drug was considered safe at lower doses (11, 13). However, in a Phase I study conducted on healthy volunteers, adverse events, including flu-like symptoms, were observed in 88.3% and 100% of the participants receiving 8 mg and 12 mg, respectively, leading to a recommendation for low-dose administration (11). The inability to administer higher doses owing to adverse effects is a major limitation of GS-9620.

For the efficacy assessment, we focused on type I IFN, a key mediator of the antiviral immune response against infected cells. Although 1 nonresponder was observed in both the 1 and 3 mg/kg SA-5 groups, a dose-dependent increase in circulating type I IFN levels was observed, indicating the immunostimulatory potential of SA-5. Supporting its clinical potential, a recent study using an HBV-infected mouse model demonstrated that the administration of SA-5 at 3 mg/kg markedly induced ISG15 through type I IFN signaling, resulting in a marked reduction in HBV DNA levels (16). In contrast, although GS-9620 has been shown to induce both type I IFN secretion and ISG15 expression in HBV-infected patients, it ultimately failed to achieve a significant reduction in HBsAg levels and, thus, did not progress to clinical application (11) . One of the limitations that may have contributed to the lack of clinical efficacy of GS-9620 is the inability to administer high doses due to adverse effects. In a Phase I study involving healthy volunteers, plasma drug levels at doses ≤ 4 mg were markedly lower than those observed at 8–12 mg, suggesting that clinical trials conducted at ≤ 4 mg in patients with chronic HBV may not have achieved sufficient therapeutic exposure (11–13, 24). These findings highlight the importance of identifying agents or dosing regimens that allow the highest possible exposure while minimizing off-target responses. In the case of SA-5, careful evaluation of the efficacy in patients is required. Notably, the induction of circulating type I IFN declined with repeated dosing at all dose levels. A similar trend was observed for the activation of pDCs, the primary producers of type I IFN (27, 28), which diminished over the course of repeated administration. This suggests that pDC desensitization contributes to the attenuation of IFN responses (28). Although frequency-based analyses at 3 mg/kg did not reach statistical significance, median values and CD80 MFI indicated activation consistent with IFN-α induction.

In addition, mDCs displayed activation patterns broadly similar to pDCs, with significant CD80 upregulation at ≥ 3 mg/kg (Supplemental Figure 10), indicating that SA-5 engages multiple antigen-presenting cell subsets with distinct magnitudes and kinetics.

However, it should be noted that the efficacy assessment in this study was limited to an indirect evaluation based on type I IFN pathway activation. As with GS-9620, direct confirmation of the clinical efficacy of SA-5 in humans is required.

Although the present study primarily focused on the safety and efficacy of SA-5, its adjuvant activity is likely mediated through TLR7 activation. This assumption is supported by structural similarity to DSP-0509, a validated TLR7 agonist, and by our own data showing concentration-dependent activation of recombinant human and cynomolgus TLR7-expressing cells, as well as functional activation of pDCs, the principal TLR7^+^ population in these species. Through this pathway, SA-5 induces type I IFN responses, a key driver of its immunostimulatory effects. Future work, including in vivo pDC depletion studies or in vitro analyses using purified immune subsets, will be valuable to more precisely delineate this mechanism. Furthermore, in comparison with GS-9620, the targeted distribution of SA-5 may favor localized pDC activation, which could contribute to reduced systemic adverse effects.

Based on our findings, we propose 3 mg/kg as the recommended dose of SA-5 to achieve a favorable balance between safety and efficacy. When comparing SA-5 and the existing TLR7 ligand GS-9620 at an equivalent dose of 1 mg/kg, SA-5 exhibited comparable efficacy, whereas GS-9620 was associated with markedly lower safety. SA-5 is designed for intestinal-specific delivery, allowing hepatic targeting via the portal vein and subsequent biliary excretion (16). This delivery mechanism likely contributes to reduced systemic exposure, which may underlie the superior safety profile of SA-5 compared with that of GS-9620 observed in our comparative study. Indeed, in a clinical trial involving 192 patients with HBV who had not received prior antiviral therapy, 65.9% experienced adverse events during 24 weeks of GS-9620 administration (24). The most common symptoms are flu-like manifestations, such as fever and myalgia, raising concerns about systemic effects. Among patients receiving 1, 2, or 4 mg doses, grade 3 adverse events, such as elevated liver enzymes and flu-like symptoms, led to treatment discontinuation in 2 patients (3.6%) at 2 mg and 4 patients (7.9%) at 4 mg. In the present study, although SA-5 demonstrated efficacy comparable with that of GS-9620, it was associated with lower CRP levels and fewer adverse responses, suggesting a reduction in systemic immune activation. Notably, SA-5 showed a comparable safety profile at both 1 and 3 mg/kg, indicating its potential for higher dosing with reduced systemic toxicity compared with GS-9620. These results suggest that SA-5 may overcome one of the primary limitations of GS-9620 — namely, the inability to administer therapeutically effective high doses owing to adverse effects.

However, given that many patients with potential target indications for SA-5, such as chronic persistent infections and cancer, are middle-aged or older, it is essential to evaluate age-related changes in efficacy and risk (29). Therefore, the assessment of SA-5 should include safety and efficacy evaluations using appropriate aging models that account for physiological aging. Among the experimental animals, NHPs are generally considered the most physiologically relevant to humans because of their close genetic similarity (30). We previously demonstrated that genes identified through gene expression profiling of liver tissues from both young and aged cynomolgus monkeys were markedly upregulated in aged human liver samples, supporting the relevance of cynomolgus monkeys as a useful model for studying liver aging (31). In addition, we have previously evaluated the safety and efficacy of STING ligands, another class of innate immune activators, in both young and aged cynomolgus monkeys (15). In the study, aged monkeys exhibited reduced safety and efficacy compared with younger animals, and an inverse correlation was observed between age and both type I IFN induction and pDC activation. In contrast, in the present study, although aged monkeys exhibited a decrease in safety compared with younger animals, an increase in efficacy was observed. Among individual animals, we identified subsets showing improved efficacy without changes in safety, as well as those with increased efficacy accompanied by decreased safety. However, no individuals were observed in whom safety declined without a corresponding increase in efficacy.

Regarding potential sex-related differences, although the number of animals analyzed was small and does not allow firm conclusions, our Supplemental data (Supplemental Figure 2) suggest that female monkeys may exhibit higher baseline levels of SA-5 compared with males. This observation warrants further investigation in future studies with larger cohorts.

In vitro studies using DCs isolated from the PBMCs of aged and young individuals have reported that stimulation with the TLR7/8 ligand R848 results in reduced type I IFN production in aged donors. These studies also demonstrated decreased expression of TLR7 in pDCs derived from aged individuals (32, 33). In contrast, in the present in vivo dosing study, both type I IFN gene expression and secretion were elevated in PBMCs from aged cynomolgus monkeys, indicating a different response profile compared with previous reports. These discrepancies underscore the importance of cautious evaluation when considering drug administration in the older population. Six animals were used per group. Given this sample size, the presence of even a single nonresponder can obscure statistically significant differences; conversely, a strong response in 1 animal may produce apparent but nonsignificant trends. Therefore, the conclusions drawn from comparisons that did not reach statistical significance should be interpreted with caution.

In conclusion, we evaluated the efficacy and safety of SA-5, a potentially novel TLR7 ligand, in a NHP model. Our findings suggest that SA-5 may offer a safer dosing profile than the existing oral TLR7 agonist GS-9620. Importantly, the methodological framework established in this study is expected to serve not only in the clinical development of SA-5 but also as a valuable approach for determining appropriate dose settings in future first-in-human trials of similar innate immune stimulators.

This study highlights the potential of SA-5 as a potentially novel TLR7 agonist with an improved safety profile over GS-9620, supported by integrated analyses combining PK, safety biomarkers, and type I IFN–related efficacy parameters. These findings not only demonstrate the feasibility of hepatic-targeted TLR7 activation but also establish a methodological framework for balanced dose selection in the preclinical setting. Notably, the identification of a 3 mg/kg dose as optimal suggests a potential translational window for future human studies.

For future research, several directions should be pursued. First, mechanistic studies are needed to elucidate the molecular basis of the attenuated type I IFN response observed with repeated dosing, including the potential desensitization of plasmacytoid DCs. Second, since aged animals exhibited distinct immunological responses to SA-5, further investigation into age-associated modulation of innate immunity could refine patient stratification and dosing strategies. Third, while this study focused primarily on innate immune responses, we also evaluated T cell activation markers such as CD69 on CD4^+^ and CD8^+^ T cells. These results were inconsistent and did not show a clear dose-dependent pattern (data not shown), so no reliable conclusions could be drawn regarding T cell activation under the present conditions. Nevertheless, murine studies have reported SA-5–induced T cell activation, suggesting that species-specific factors or methodological limitations may explain the discrepancy. Further optimization of experimental conditions may therefore be required to clarify whether SA-5 can also induce adaptive T cell responses in primates. Finally, clinical studies will be essential to determine whether the safety-efficacy balance observed in NHPs translates to humans, particularly in populations with chronic viral infections or cancer. The composite biomarker and PCA-based analytical strategy presented here may also be applicable for early-stage clinical decision-making in the development of innate immune modulators.

## Methods

### Sex as a biological variable.

Our study examined male and female animals, and similar findings are reported for both sexes for Cohort 1 and 2. However, owing to the limited sample size, sex-stratified analyses were not performed.

### Animal experiments, PBMC sampling, and hematologic analysis.

For Cohort 1, eight cynomolgus macaques (4 males and 4 females, 2–3 years of age) were randomly assigned to 4 groups (*n* = 1 male and 1 female per group). Each animal received a single oral dose of SA-5 at 30 mg/kg, 100 mg/kg, 300 mg/kg, or 1,000 mg/kg.

For Cohort 2, sixteen cynomolgus macaques (8 males and 8 females, 2–3 years of age) were randomly assigned to 4 groups (*n* = 2 males and 2 females per group). Animals received SA-5 at doses of 0 mg/kg (vehicle control: 0.5% w/v methylcellulose solution), 10 mg/kg, 100 mg/kg, or 1,000 mg/kg. Dosing was performed once weekly for 4 weeks, for a total of 5 administrations.

In these cohorts, blood samples were collected at 1, 2, 4, 6, 8, 24, 48, and 72 h after administration using syringes and needles pretreated with dipotassium ethylenediaminetetraacetic acid (EDTA-2K) for PK and cytokine analyses. Plasma was separated from whole blood and promptly frozen, then stored in an ultra–low temperature freezer until analysis.

Plasma concentrations of SA-5 were determined using a liquid chromatography–tandem mass spectrometry (LC-MS/MS). Plasma samples were prepared by protein precipitation and injected into the LC-MS/MS. The PK parameters such as C_max_, T_max_, and AUC_0–t_ were obtained using noncompartmental analysis with Phoenix WinNonlin software (version 6.4; Certara INC., Radnor, PA, USA). Cytokine levels were measured using electrochemiluminescence (ECL) and Luminex-based assays. IP-10, TNF-α, and IL-6 were quantified using the U-PLEX Biomarker Group 1 (NHP) kit (Meso Scale Diagnostics, LLC., Rockville, MD, USA), while IFN-α was measured using the IFN-alpha Monkey ProcartaPlex Simplex kit (Thermo Fisher Scientific Inc., Waltham, MA, USA).

For Cohort 3, Thirty-six cynomolgus macaques aged 4–20 years were divided into 6 groups (1 mg/kg [*n* = 6], 3 mg/kg [young *n* = 6, aged *n* = 6], 5 mg/kg [*n* = 6], and 10 mg/kg [*n* = 6] for SA-5, and 1 mg/kg [*n* = 6] for GS-9620; Supplemental Table 3). Macaques were orally administered increasing doses of either SA-5 or GS-9620 at 1-week intervals.

PBMCs and plasma were isolated from blood samples collected at each time point in tubes containing EDTA using Ficoll-Paque PLUS (GE Healthcare, Buckinghamshire, UK). Purified PBMCs were incubated in fetal bovine serum (FBS; Sigma-Aldrich, St. Louis, MO, USA) containing 10% DMSO (Sigma-Aldrich, St. Louis, MO, USA) and stored in liquid nitrogen until analysis. Complete blood counts were performed using freshly isolated blood using an automated hematology analyzer (Sysmex K-4500; Sysmex, Hyogo, Japan).

### ELISA assay.

For Cohort 3, plasma was obtained from the blood supernatant during PBMC purification and cryopreserved until further use. Plasma cytokine levels were analyzed using ELISA kits purchased from MABTECH (Nacka Strand, Sweden) for evaluating human IL-6 (3460-1H-6), human IFN-α (3425-1H-6), and monkey TNF (3512M-1H-6) according to the manufacturer’s protocol. The ELISA signals were detected using an Epoch 2 Microplate Spectrophotometer (BioTek).

### qPCR.

mRNA was extracted from PBMCs using an RNeasy Mini Kit from PBMC (Qiagen, Hilden, Germany). To synthesize cDNA, isolated mRNA was reverse transcribed using the SuperScript III First-Stand Synthesis System (Invitrogen). qPCR was performed using HUNDERBIRD Probe qPCR Mix (TOYOBO, Japan), RT enzyme (Invitrogen, Thermo Fisher Scientific), ROX (Invitrogen, Thermo Fisher Scientific), TaqMan probes for candidate genes (CCL3, Rh02788104_gH; CXCL11, Rh02621763_m1; Eukaryotic 18S rRNA_ Hs99999901_s1; IFIT1, Rh00929909_m1; IFNA1, Hs 00256882_s1; IFNB1, Rh02913347_s1; IL1B, Rh02621711_m1; IL6, Rh02621719_u1; IL23A, Rh02872166_m1; IRF7, Rh02839174_m1; ISG15, Rh02915441_g1; MX1, Rh02842279_m1; TNF, Rh02789784_m1), and a reference gene (GAPDH, Forward, 5′-AGAAGTATACAACAGCCTCA-3′; Reverse, 5′-ACTGTGGTCATGAGTCCTTC-3′; FAM, 5′-ACCACCAACTGCTTAGCACC-3′), with StepOne Plus Real-time PCR system. The following thermal cycling conditions were used: 30 min at 45°C, 5 min at 95°C, 40 cycles of 1 s at 95°C, 30 s at 54°C, and 30 s at 60°C for MX1, and CXCL11, 40 cycles of 1 s at 95°C, and 60 s at 60°C for others.

### Flow cytometric analysis.

Frozen PBMCs were thawed and washed with Roswell Park Memorial Institute 1640 medium (Sigma) supplemented with 10% FBS (Sigma), 100 unit/mL penicillin, and 100 mg/mL streptomycin (Sigma, the supplemented medium hereafter R10). The cells were treated with 1 mL of 50 U/mL benzonase (Merck, Darmstadt, Germany) in R10 for 60 min at 37°C. After incubation, the cells were stained using a Fixable Viability Stain 440UV kit (FVS440; Becton, Dickinson, and Co. Franklin Lakes, NJ, USA; BD) at room temperature (RT) for 5 min at RT. The CC-chemokine receptor 7 (CCR7) was stained at 37°C for 10 min, and the antibodies for the remaining markers listed in Supplemental Table 4 were added and incubated at RT for 15 min. After staining, cells were washed twice with PBS, fixed with 1% paraformaldehyde, and analyzed using the FACSymphony A5SE flow cytometer (BD) equipped with 5 lasers.

### Flow cytometry data processing for safety and efficacy evaluation of individual macaques.

FCS files were analyzed using FlowJo software (version 10.10.0; Becton, Dickinson, and Co., Franklin Lakes, NJ, USA). Plots were generated using R software version 4.1.0. by using the listed packages.

### Multiparameter analysis for safety and efficacy evaluation of individual macaques.

The experimentally obtained raw data were preprocessed as follows. First, a minimum threshold value was set; if the minimum value of a parameter was less than zero, the threshold was set to zero. For the fold-change calculation, if the baseline value was zero, the nearest nonzero value was extracted and used as the baseline value.

To align the postadministration time points (0, 1, and 7 d) for each administration, we selected 4 administration points: Ad.01, Ad.02, Ad.07, and Ad.12; d0 of the next administration was set to be the same as d7 of the previous administration (e.g., d7 of Ad.01 and d0 of Ad.02 refer to the same data). The FC values were calculated relative to d0 for each administration and d0 for the first administration.

To score the outlier values in complete blood count and biochemical tests, we first selected 35 parameters for which standard ranges were available. We calculated the median value of each standard range and set 2 thresholds: tier 1 thresholds were defined as the upper limit plus half of the median value and the lower limit minus half of the median value, and tier 2 thresholds were defined as the upper limit plus the full median value and the lower limit minus the full median value. If the actual value exceeded the standard range, a score of +1 was assigned. If the score exceeded the Tier 1 threshold, a score of +2 was assigned. If it exceeded the Tier 2 threshold, a score of +4 was assigned. The total score was defined as the CBC score for outlier evaluation. Consequently, the outlier levels were categorized as follows: 0, within range; 1 = weak outlier; 3 = moderate outlier; and 7 = strong outlier.

PCA was performed using the *prcomp* function in the R statistical package. The PCA plots for safety and efficacy were generated separately using the selected parameters, as schematically shown in Figure 2A and Figure 4A, respectively. Briefly, for the safety PCA, the parameters associated with dose-dependent differences were selected as follows. The experimental groups were further divided into 2 groups based on the dose levels: 1–3 mg/kg and 5–10 mg/kg. The following parameters were compared: (a) actual values and fold-change values 1 d after the first administration (Ad.01d1/Ad.01d0), (b) fold-change values relative to the baseline value prior to the first administration across all dosing points, and (c) deviation scores from reference values. We compared the intergroup differences using the Wilcoxon test and extracted 27 parameters that showed statistically significant differences. For efficacy PCA, we manually selected 10 parameters known to be involved in type I IFN signaling.

To create a plot that simultaneously shows the analysis for safety and efficacy, PC1 values from the safety and efficacy PCA data were combined and plotted in a 2-dimensional space. Individual macaques were classified into 4 groups based on PC1 values for safety and efficacy, with the threshold defined by the maximum value of the ellipse.

### Statistics.

Statistical analyses were performed using R/Bioconductor (R version 4.4.2) or GraphPad Prism software (version 8.3.0; GraphPad Software). The experimental variables were compared using the nonparametric Wilcoxon/Mann-Whitney *U* test unless otherwise stated. For analyses comparing dose groups across multiple administrations, the Wilcoxon rank-sum test stratified by administration (van Elteren test) was used. When multiple comparisons were performed, *P* values were adjusted using the Benjamini-Hochberg FDR method, and adjusted *q* values were reported. Statistical significance was defined as *P* < 0.05 for single comparisons and FDR-adjusted *q* < 0.05 for analyses involving multiple comparisons, unless otherwise noted. For the cytokine correlation data, each cytokine concentration in the plasma was scaled, variances were adjusted to 1 within parameters using the scale function of the R scales package, and Spearman’s correlation values were calculated.

### Study approval.

Cynomolgus macaques (Macaca fascicularis) used in Cohorts 1 and 2 were confirmed to be negative for simian immunodeficiency virus (SIV), simian type D retrovirus, simian T cell leukemia virus, B virus, and filoviruses. All animals were bred at NAFOVANNY (Dong Nai, Vietnam) and supplied by Eve Bioscience (Wakayama, Japan). Animal studies were performed at the SNBL INA Ltd. (Nagano, Japan), an AAALAC International-accredited facility. All procedures were approved by the IACUC of SNBL INA Ltd. (approval no. 22066 for Cohort 1 and no. 22115 for Cohort 2). Animals were housed and monitored under the supervision of the veterinarian. For Cohort 3, cynomolgus macaques, all of which were negative for SIV, simian type D retrovirus, simian T cell lymphotropic virus, simian foamy virus, Epstein-Barr virus, cytomegalovirus, and B virus — housed at the Tsukuba Primate Research Center (TPRC), National Institutes of Biomedical Innovation, Health, and Nutrition (NIBN) — were used. Animal studies were performed at NIBN with the approval of the institutional Committee on the Ethics of Animal Experiments of NIBN (approval no. DSR03-22). The animals were supervised by the veterinarians in charge of the animal facility.

### Data availability.

The data generated and/or analyzed in this study are available from the corresponding author upon reasonable request. Values for all data points in graphs are reported in the Supporting Data Values file.

## Author contributions

ST contributed conceptualization, methodology, investigation, formal analysis, visualization, writing of the original draft, and funding acquisition. TT contributed investigation, formal analysis, visualization, and writing of the original draft. YN, HM, and TN contributed investigation and resources. SY and TK contributed supervision, conceptualization, and review and editing of the manuscript. MK, SO, HK, and AF contributed resources and investigation (compound synthesis and PK). TY contributed conceptualization, supervision, project administration, writing the original draft, writing the review and editing, and funding acquisition. All authors reviewed and edited the manuscrip and approved the final version. ST and TT contributed equally to this work. Both authors were involved in the design, execution, and analysis of the experiments and cowrote the original draft. The order of authorship among co–first authors was determined based on the extent of their contributions to experimental design, data analysis, and manuscript preparation.

## Funding support

- The Japan Society for the Promotion of Science/Scientific Research (B) (grant no. JP20H03728).
- The Japan Agency for Medical Research and Development (grant no. JP25fk0310542).

## Supplementary Material

Supplemental data

Supporting data values

## Version 1 11/11/2025

In-Press Preview

## Version 2 12/22/2025

Electronic publication
