# Supplementary material for: Preclinical assessment of oral TLR7 agonist SA-5 in a nonhuman primate model
Source: JCI Insight. 2025 Nov 11;10(24):e196809. doi: 10.1172/jci.insight.196809 (PMC12890520; doi:10.1172/jci.insight.196809)
Supplement: Supplemental data [file jciinsight-10-196809-s196.pdf]

Supplemental Figure

Supplemental Figure S1

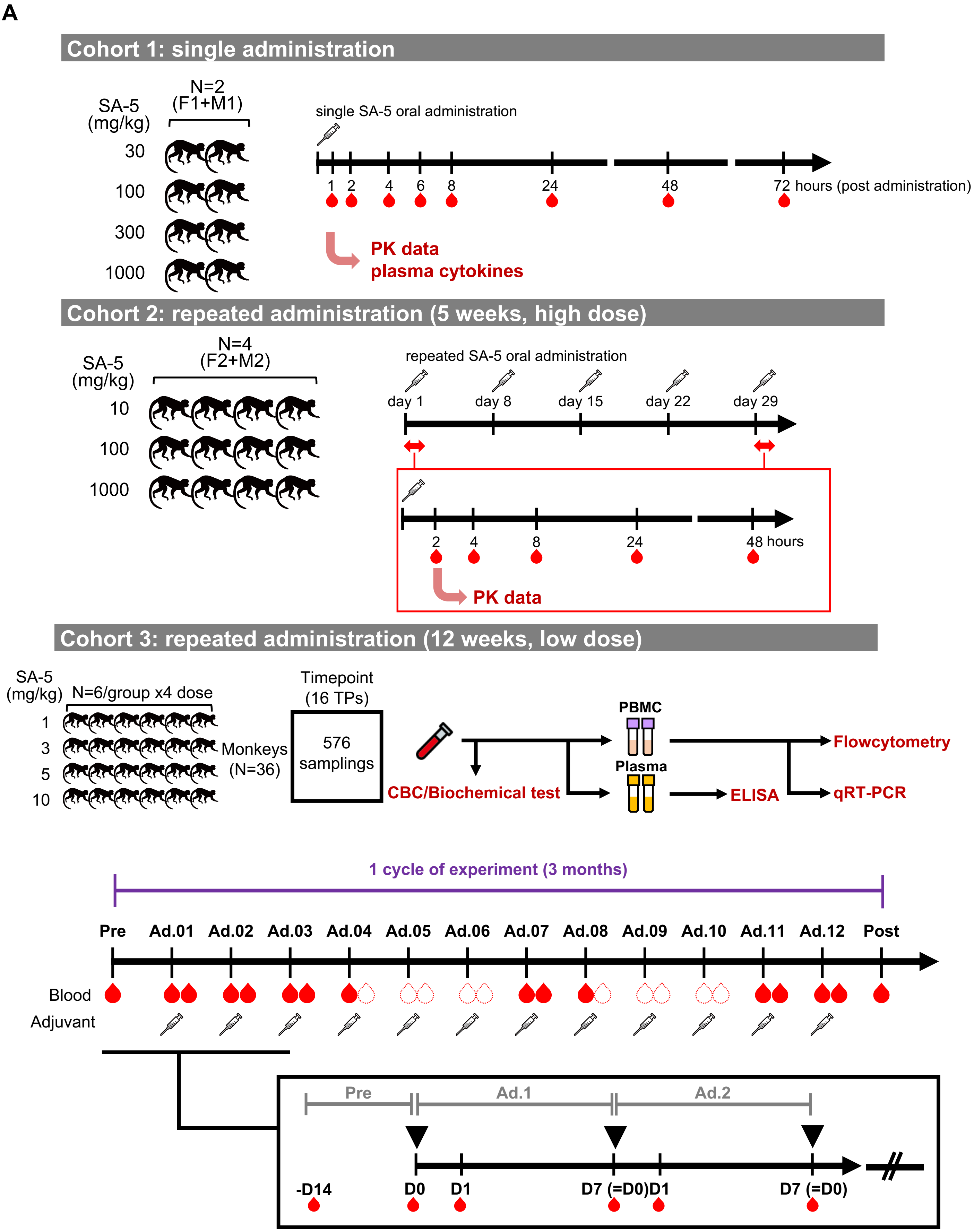

Supplemental Figure S1

Supplemental Figure S1.
Schematic depiction of each cohort used in this study
A)
• Cohort 1: Single oral administration of SA-5 at 30, 100, 300, or 1000 mg/kg (n = 2 per group; 1 male and 1 female). Blood samples were collected at multiple time points up to 72 hours for pharmacokinetic (PK) and plasma cytokine analyses.
• Cohort 2: Repeated high-dose oral administration of SA-5 at 10, 100, or 1000 mg/kg once weekly for 5 weeks (n = 4 per group; 2 males and 2 females). PK analysis was performed on day1 and day 29 at the indicated time points.
• Cohort 3: Repeated low-dose oral administration of SA-5 at 1, 3, 5, or 10 mg/kg weekly for 12 weeks (n = 6 per group; total n = 36). Blood was collected at 16 time points (576 samples total) for complete blood count (CBC), biochemical tests, ELISA, qRT-PCR, and flow cytometry analyses.

Sample collection, adjuvant administration, and data acquisition time points are indicated for each cohort.
Pre- and post-treatment phases and reference time points for key comparisons (e.g., day 0 [D0], day 1 [D1], and day 7 [D7]) are shown at the bottom of the schematic.

Abbreviations: CBC, complete blood count; D0/D1/D7, day 0/day 1/day 7; ELISA, enzyme-linked immunosorbent assay; PK, pharmacokinetics; qRT-PCR, quantitative reverse transcription polymerase chain reaction.

Supplemental Figure S2

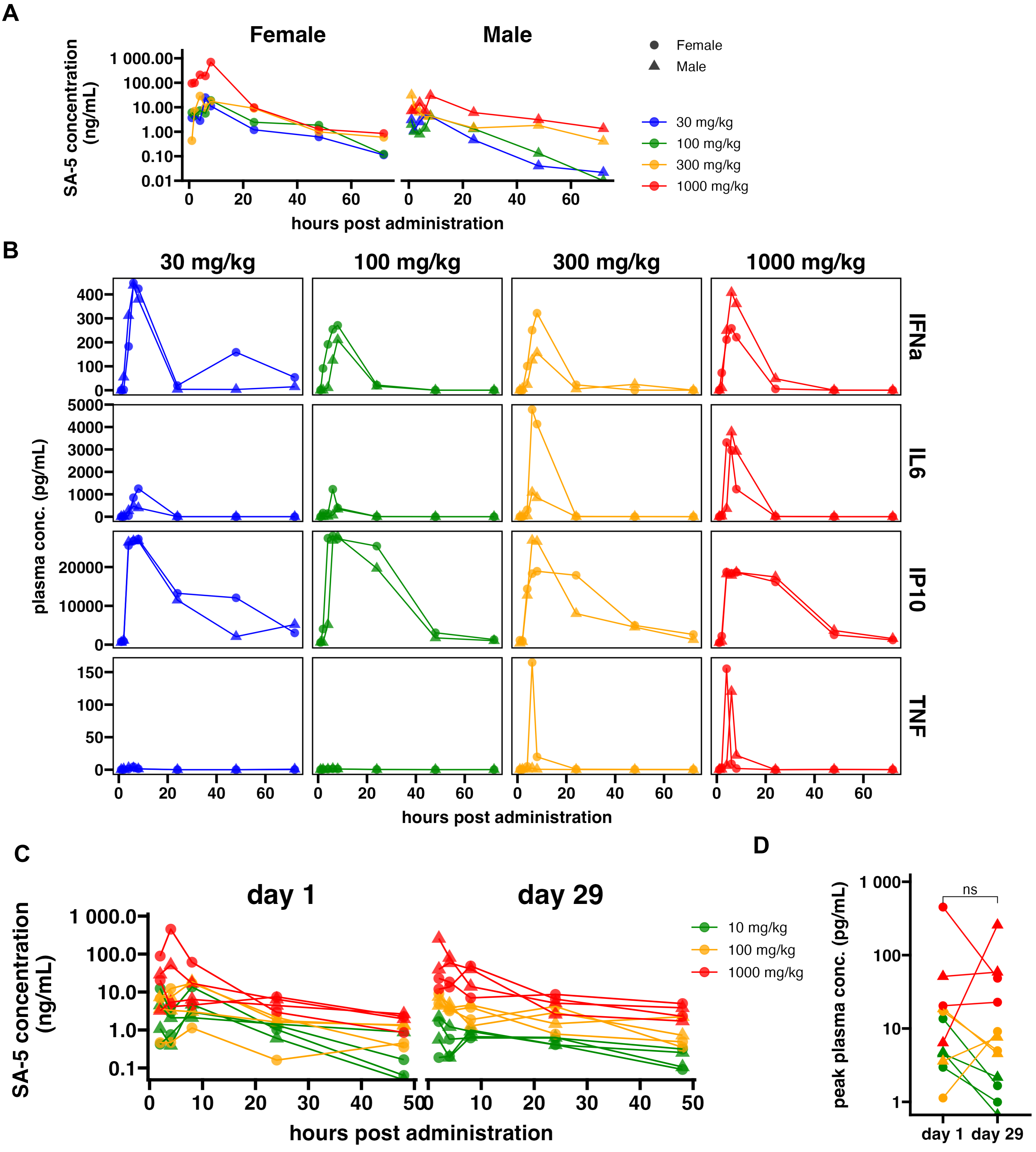

Supplemental Figure S2.
PK and plasma cytokine concentration in cohort 1 and cohort 2
A) Plasma SA-5 concentration after single oral administration of SA-5 (cohort 1). Symbols and colors indicate sex and dose.
B) Plasma cytokine concentrations after single administration of SA-5 (cohort 1), measured by ELISA. Columns represent the dose groups, and rows represent target cytokines. Symbols and colors indicate sex and dose as in (A).
C) Plasma SA-5 concentration after repeated oral administrations on day 1 and day 29 in cohort 2. Symbols and colors indicate sex and dose.
D) Comparison of peak plasma SA-5 concentrations after administration on day 1 and day 29. Statistical significance were determined using the paired Mann-Whitney *U* test (ns, not significant).

Abbreviations: ELISA, enzyme-linked immunosorbent assay; PK, pharmacokinetics; ns, not significant.

Supplemental Figure S3

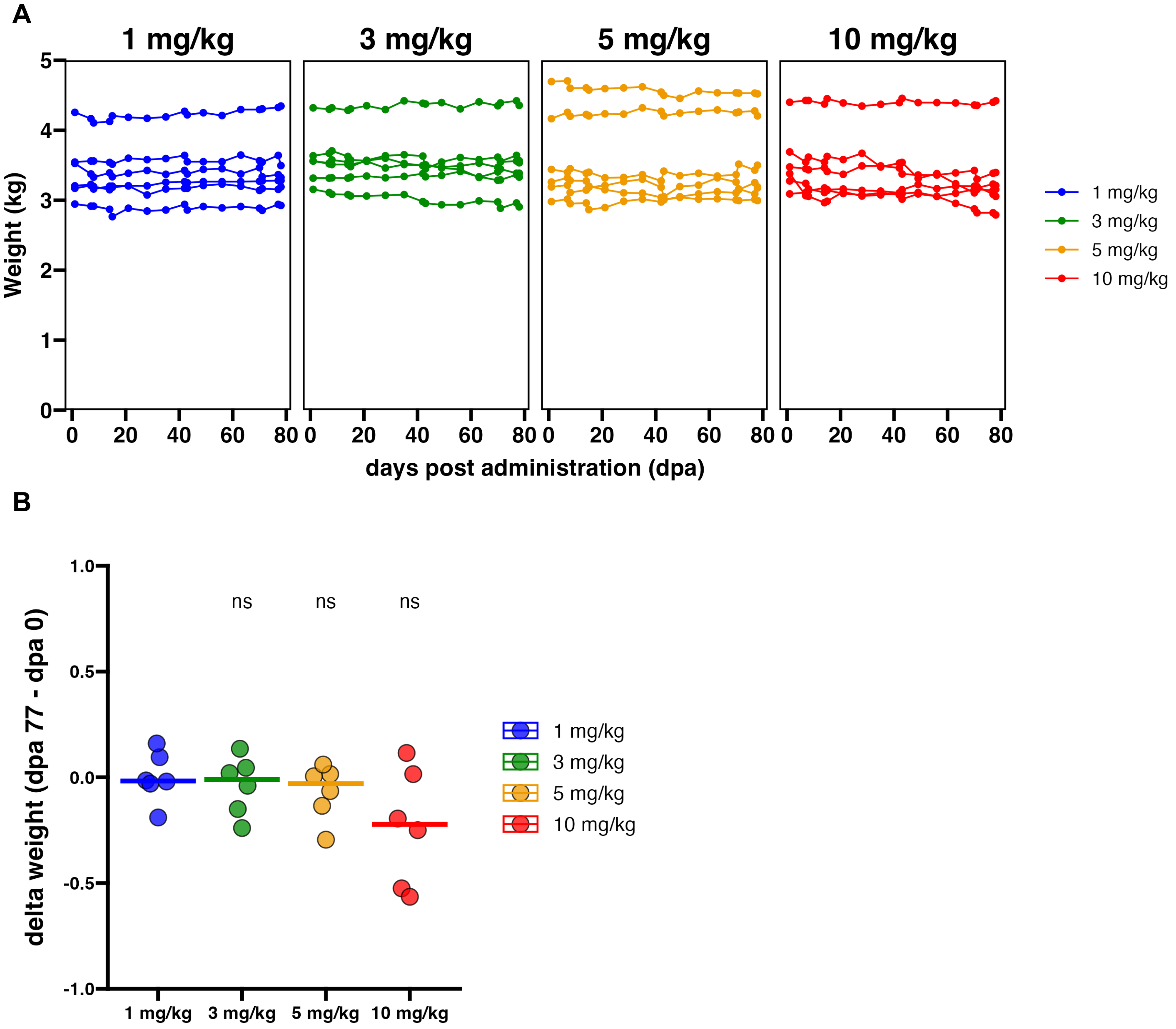

Supplemental Figure S3.
Body weight changes during repeated oral administration of SA-5 in cynomolgus macaques
(A) Line plots indicate individual body weight trajectories during repeated administration of SA-5 at the indicated doses (1, 3, 5, 10 mg/kg). Body weight was monitored across 12 dosing sessions. Thin lines represent individual animals; bold lines represent group medians. No substantial weight loss was observed throughout the study period. (B) Dot plot indicates the change in body weight (Δ weight: dpa 77 – dpa 0) for each animal across groups. Horizontal bars indicate the group medians. Statistical significance relative to 1 mg/kg were determined using the Mann-Whitney *U* test. No statistically significant differences were observed between groups (ns, not significant).

Abbreviations: dpa, days post administration; ns, not significant.

Supplemental Figure S4

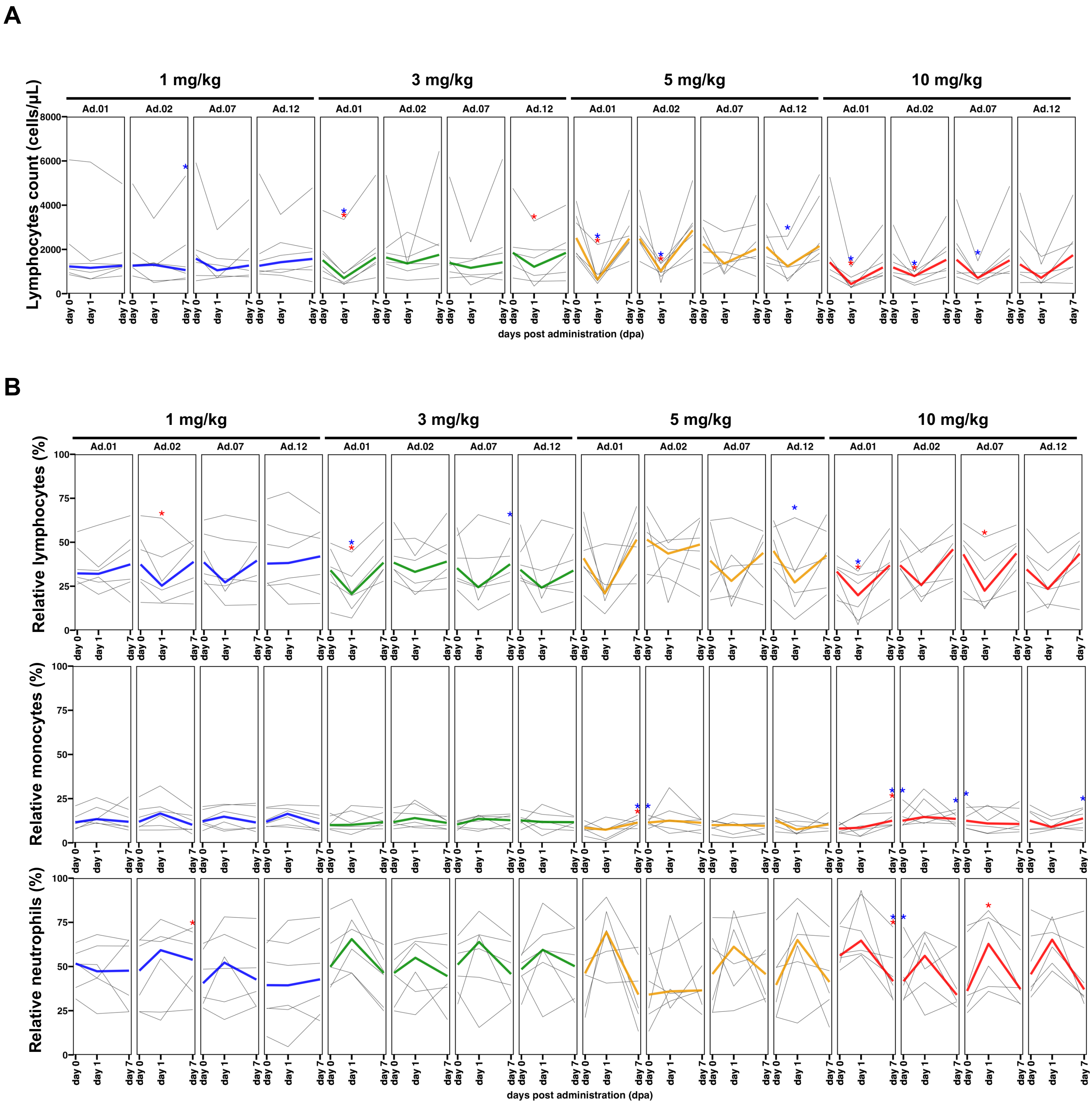

Supplemental Figure S4

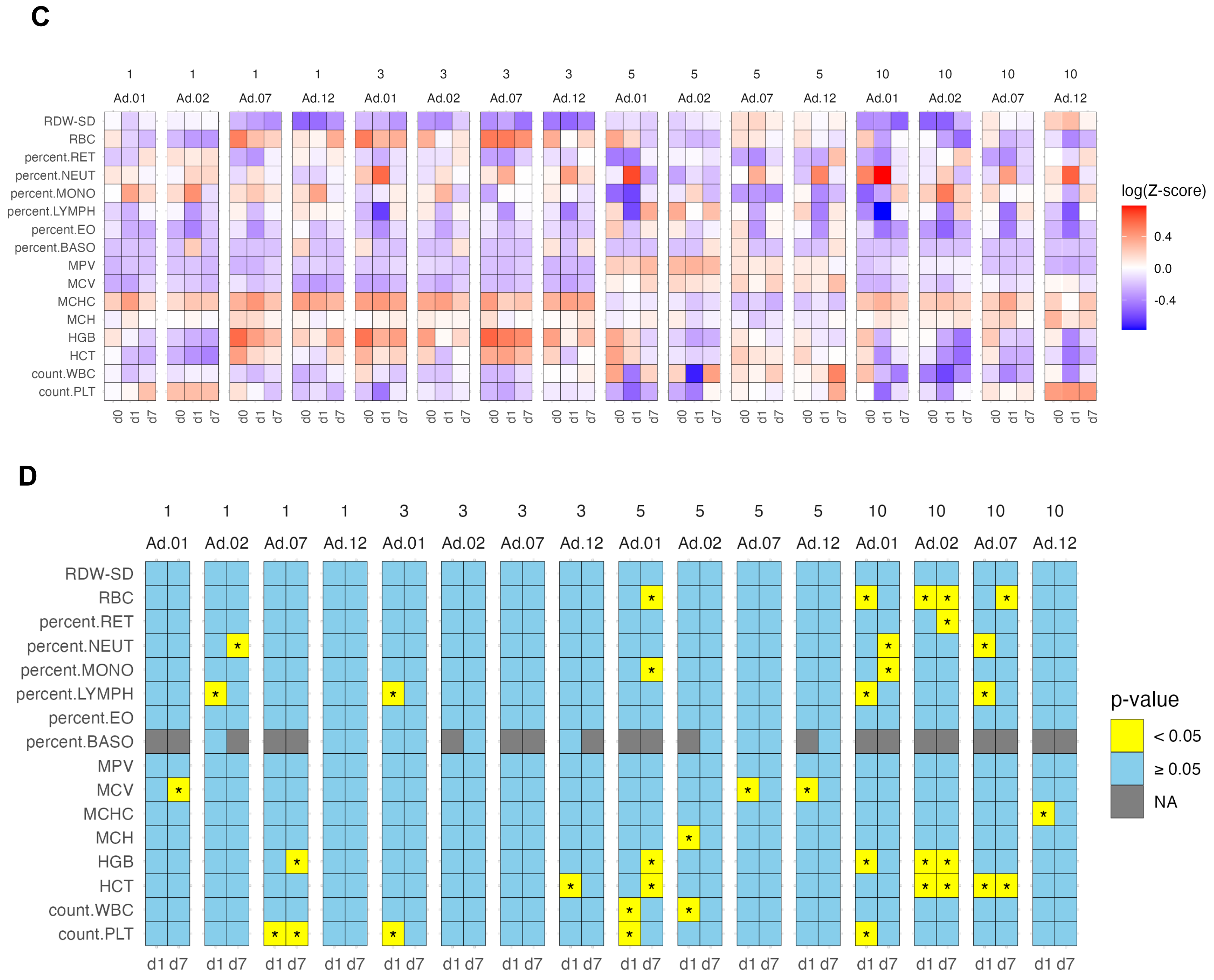

Supplemental Figure S4.
Hematological analysis following repeated administration of SA-5 in cynomolgus macaques.
A) Line plots indicate absolute lymphocyte counts (cells/μL) measured by complete blood count (CBC) at day 0 (d0), day 1 (d1), and day 7 (d7) after each administration of SA-5 at the indicated doses.
B) Line plots indicate the relative proportions (%) of lymphocytes (top), monocytes (middle), and neutrophils (bottom) in peripheral blood at the same time points.
C) Heatmap of hematological parameters visualized using log-transformed z-scores. Each column represents an individual animal per time point and administration; each row represents a specific hematological parameter.
D) Summary of p-values for comparisons between day 0 and day 1/day 7. Yellow indicates $p < 0.05$, blue indicates $p \geq 0.05$, and gray indicates not available (NA). Rows correspond to hematological parameters; columns indicate time points and dose groups

(A, B) In all panels, bold lines represent group medians, and thin lines represent individual macaques. Colors indicate SA-5 dose groups (1, 3, 5, and 10 mg/kg). Statistical significance was determined using the paired Mann–Whitney *U* test for comparisons with day 0. Blue asterisks indicate comparisons with day 0 of Administration 1 (Ad.01), and red asterisks indicate comparisons with day 0 of each corresponding administration. (*$p < 0.05$, **$p < 0.01$). (D) Statistical significance was determined using the paired Mann–Whitney *U* test for comparisons with day 0.

Abbreviations: CBC, complete blood count; NA, not available, d0/d1/d7, day 0/day 1/day 7.

Supplemental Figure S5

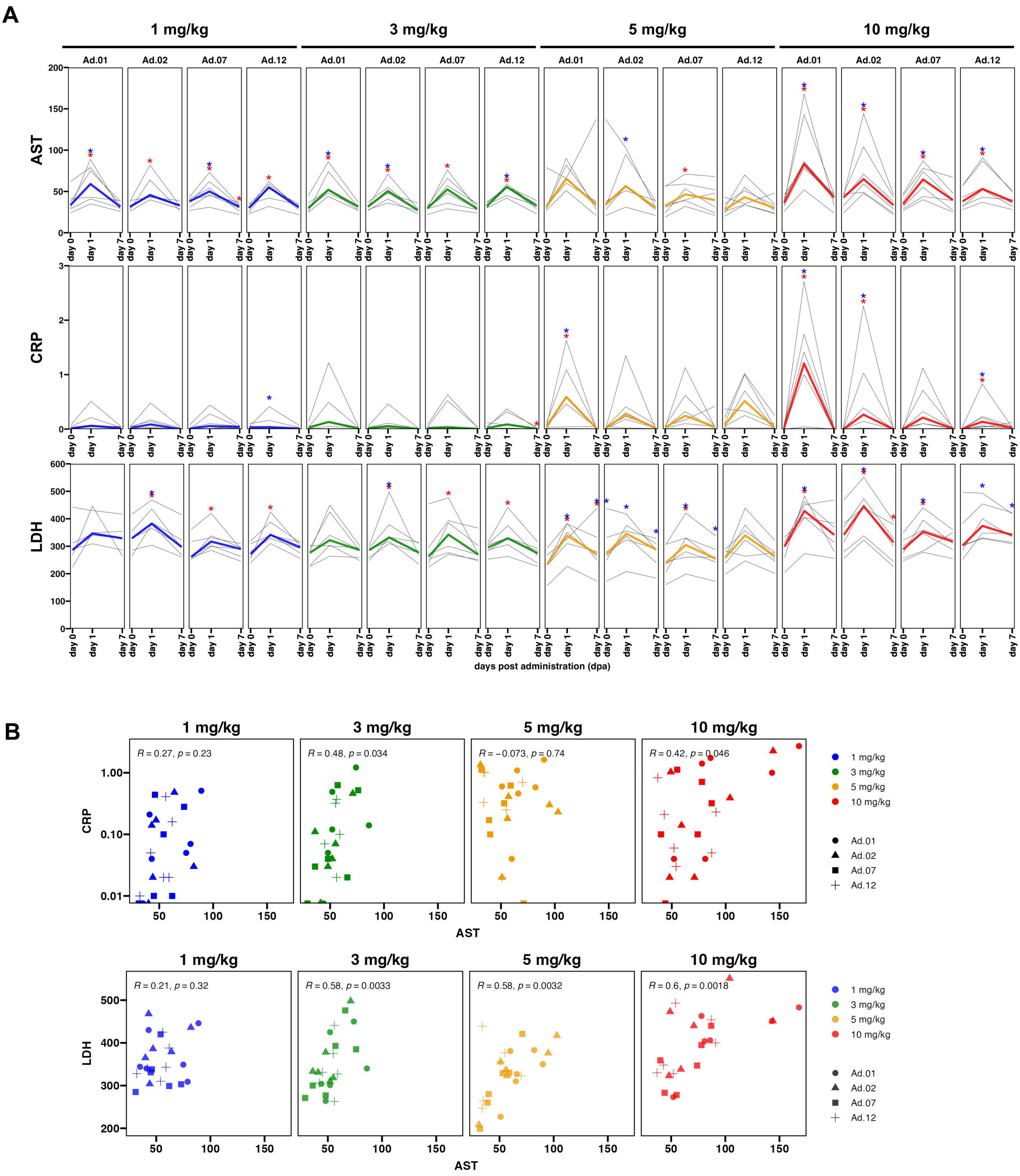

Supplemental Figure S5

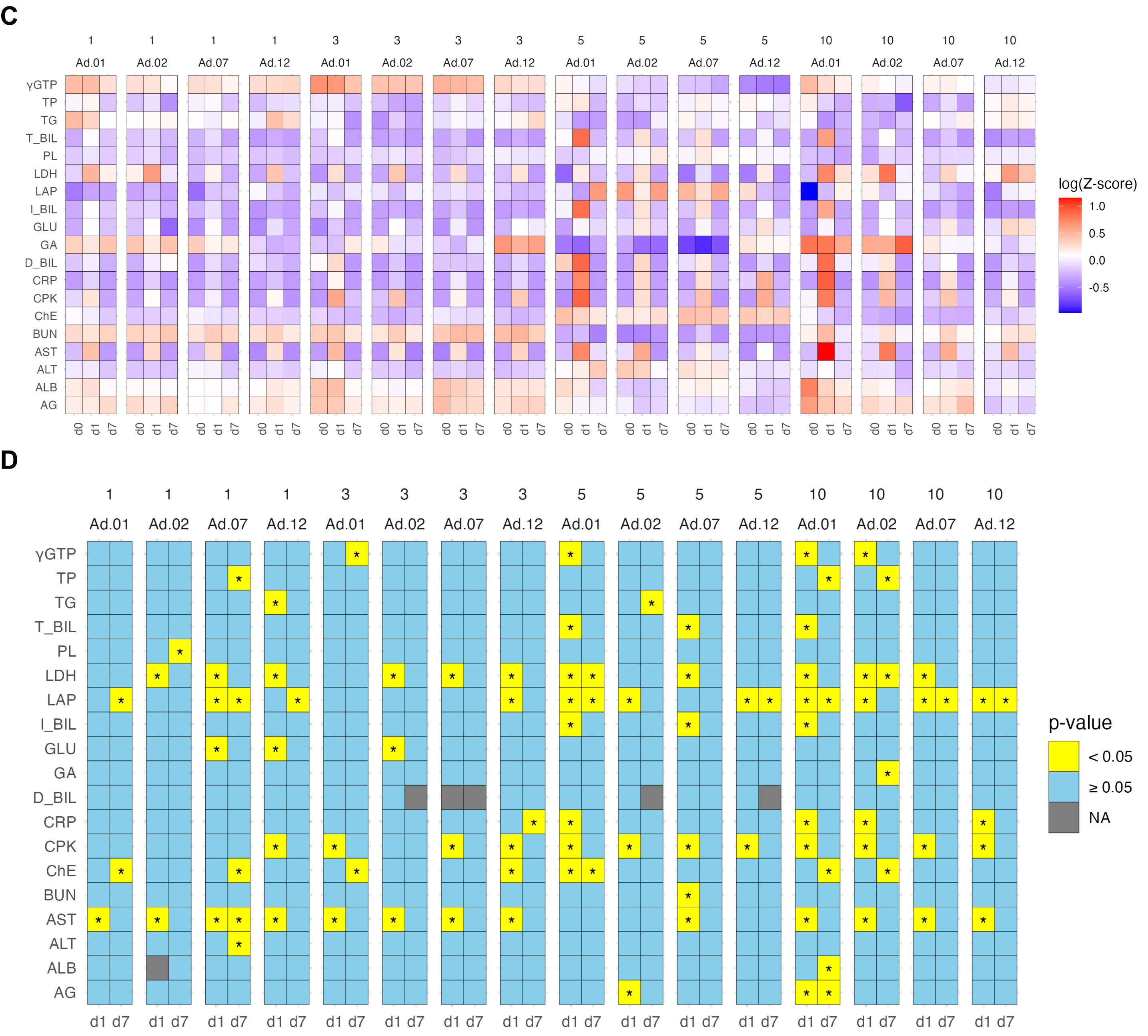

Supplemental Figure S5
Safety assessment of multiple SA-5 administration by different dose in cynomolgus macaque
A) Line plots indicate plasma levels of aspartate aminotransferase (AST; top), C-reactive protein (CRP; middle), and lactate dehydrogenase (LDH; bottom) at day 0, day 1, and day 7 following each administration (Ad.01 to Ad.12). Colors indicate dose groups (1, 3, 5, and 10 mg/kg). Bold lines represent group medians, and thin lines represent individual animals.
B) Scatter plots indicate correlation analyses between AST and CRP (top), or LDH (bottom), on day 1 after each administration. Each panel represents a different dose group. Symbols indicate administration number (circle: Ad.01, triangle: Ad.02, square: Ad.07, cross: Ad.12). Spearman's correlation coefficients (R) and p-values are shown.
C) Heatmap of clinical chemistry parameters visualized using log-transformed z-scores. Each column represents an individual animal per time point and administration; each row represents a specific parameter.
D) Summary of *p*-values for comparisons between day 0 and day 1/day 7. Yellow indicates $p < 0.05$, blue indicates $p \geq 0.05$, and gray indicates not available (NA).

(A) In all panels, bold lines represent group medians, and thin lines represent individual macaques. Colors indicate SA-5 dose groups (1, 3, 5, and 10 mg/kg). Statistical significance was determined using the paired Mann–Whitney *U* test for comparisons with day 0. Blue asterisks indicate comparisons with day 0 of Administration 1 (Ad.01), and red asterisks indicate comparisons with day 0 of each corresponding administration. (*$p < 0.05$, **$p < 0.01$). (B) Statistical significance was determined using the Spearman's rank correlation test. (D) Statistical significance was determined using the paired Mann–Whitney *U* test for comparisons with day 0.

Abbreviations: AST, aspartate aminotransferase; CRP, C-reactive protein; LDH, lactate dehydrogenase; NA, not available; log1p, log(x+1) transformation; Ad., administration.

Supplemental Figure S6

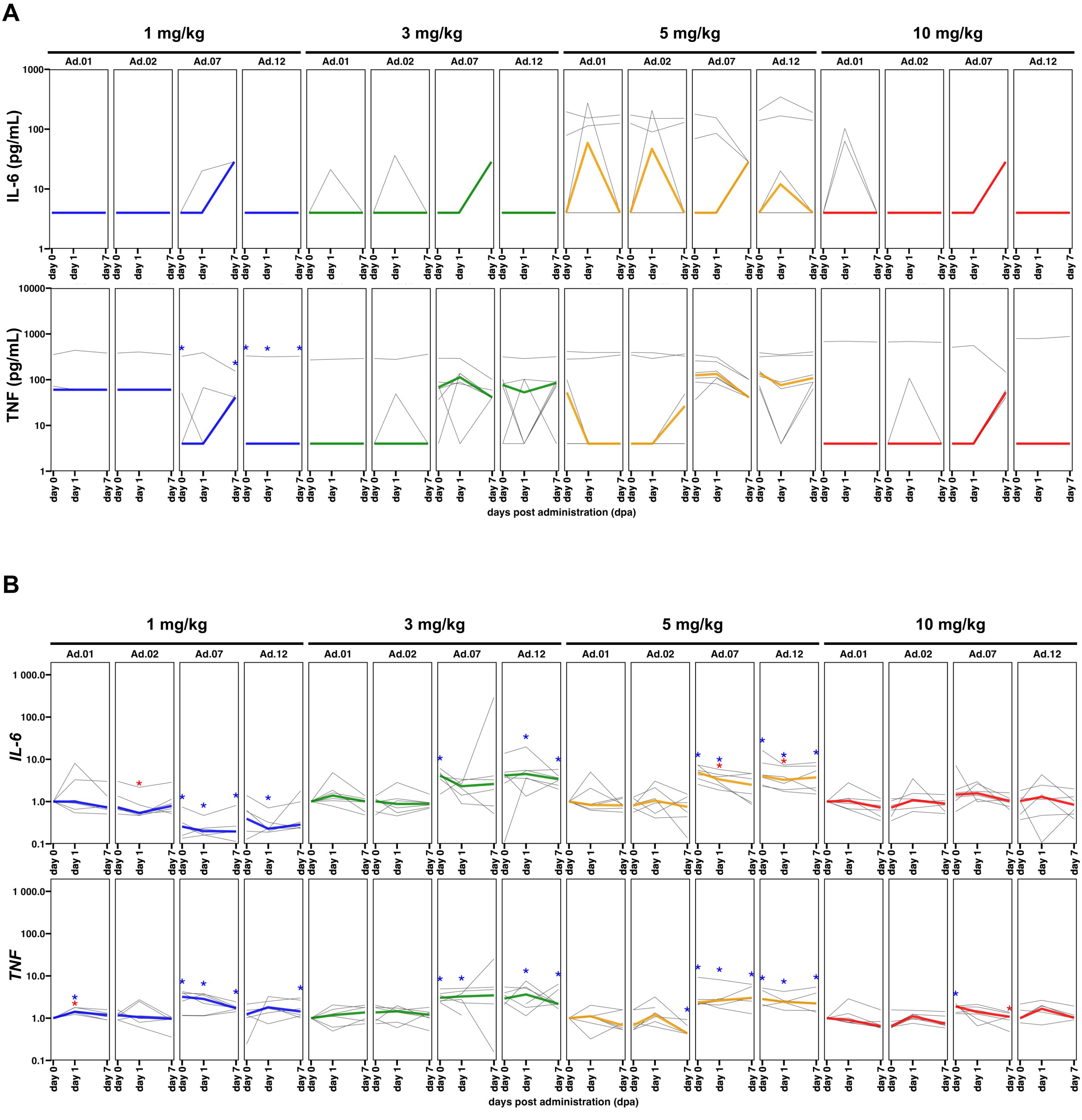

Supplemental Figure S6.
Longitudinal assessment of IL-6 and TNF responses following repeated administration of SA-5 in cynomolgus macaques
A) Line plots indicate plasma concentrations of interleukin-6 (IL-6; top) and tumor necrosis factor (TNF; bottom) measured by ELISA at day 0, day 1, and day 7 after each administration (Ad.01 to Ad.12). Values are shown on a logarithmic scale.
B) Line plots indicate relative mRNA expression levels of IL-6 (top) and TNF (bottom) in peripheral blood mononuclear cells (PBMCs) at the same time points, measured by quantitative reverse transcription PCR (qRT-PCR). Expression levels are normalized and plotted on a log scale.

(A,B) In all panels, bold lines represent group medians, and thin lines represent individual macaques. Colors indicate SA-5 dose groups (1, 3, 5, and 10 mg/kg). Statistical significance was determined using the paired Mann–Whitney *U* test for comparisons with day 0. Blue asterisks indicate comparisons with day 0 of Administration 1 (Ad.01), and red asterisks indicate comparisons with day 0 of each corresponding administration. (**p* < 0.05 , ***p* < 0.01).

****Abbreviations**:** IL-6, interleukin-6; TNF, tumor necrosis factor; PBMC, peripheral blood mononuclear cell; qRT-PCR, quantitative reverse transcription polymerase chain reaction.

Supplemental Figure S7

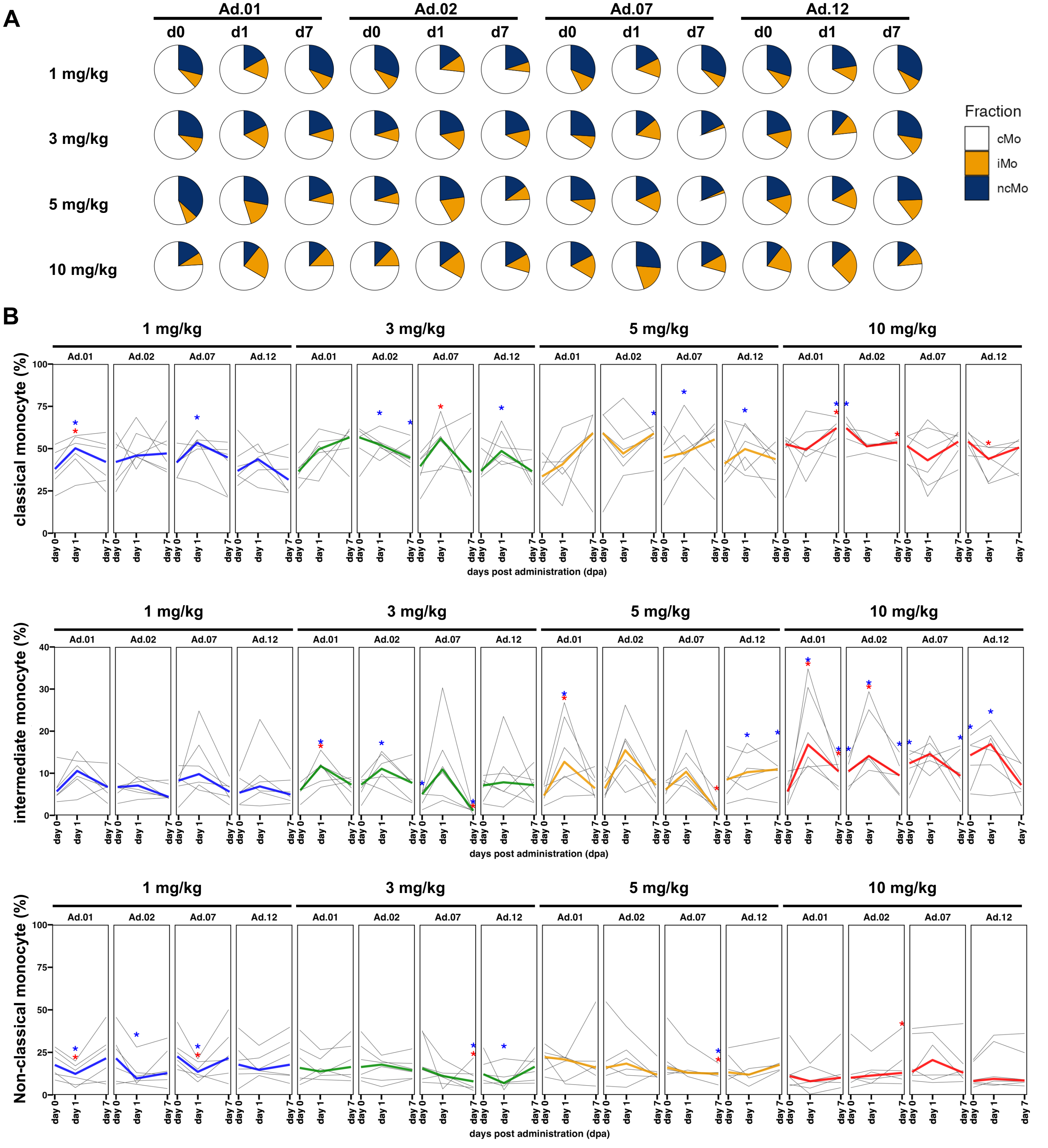

Supplemental Figure S7

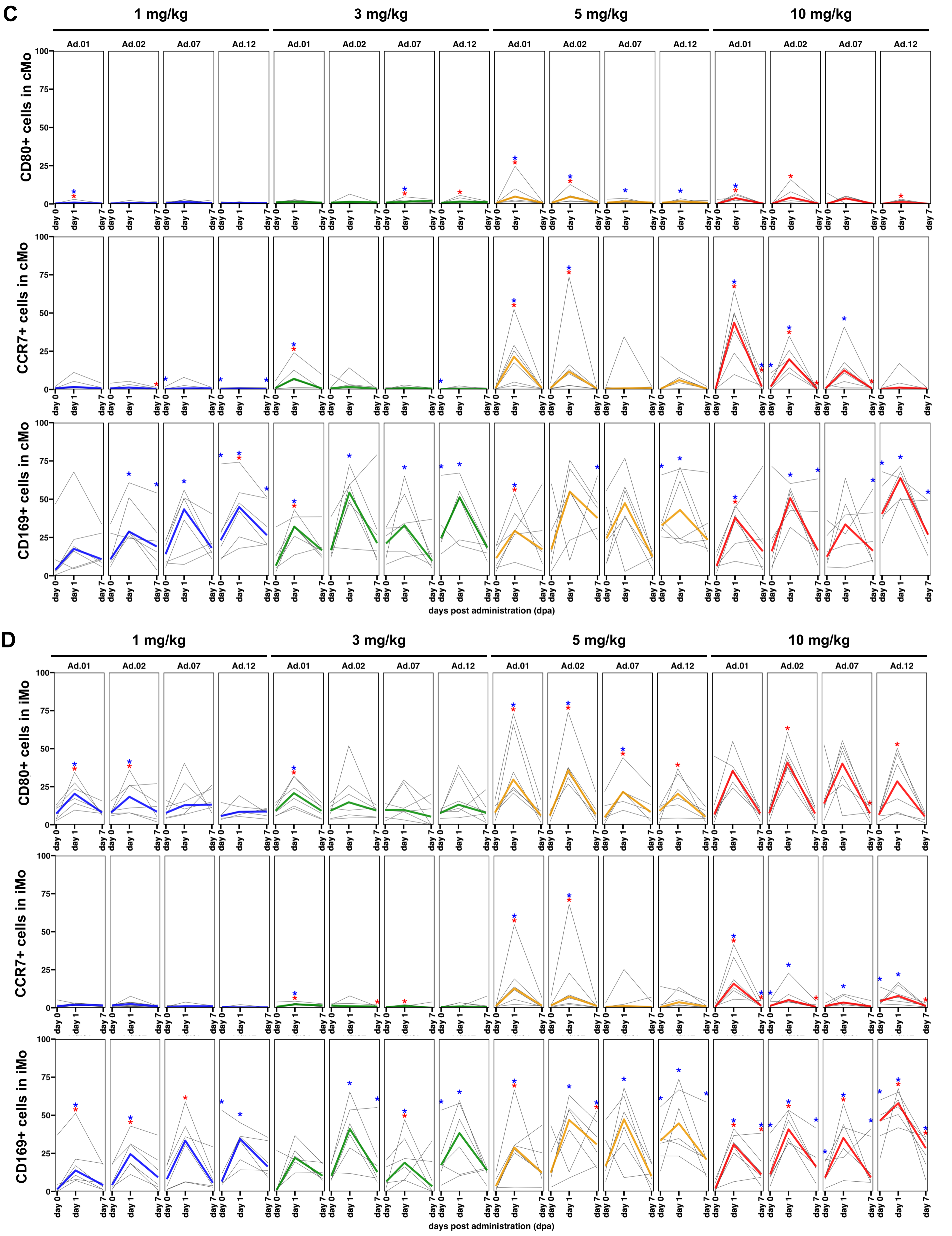

Supplemental Figure S7

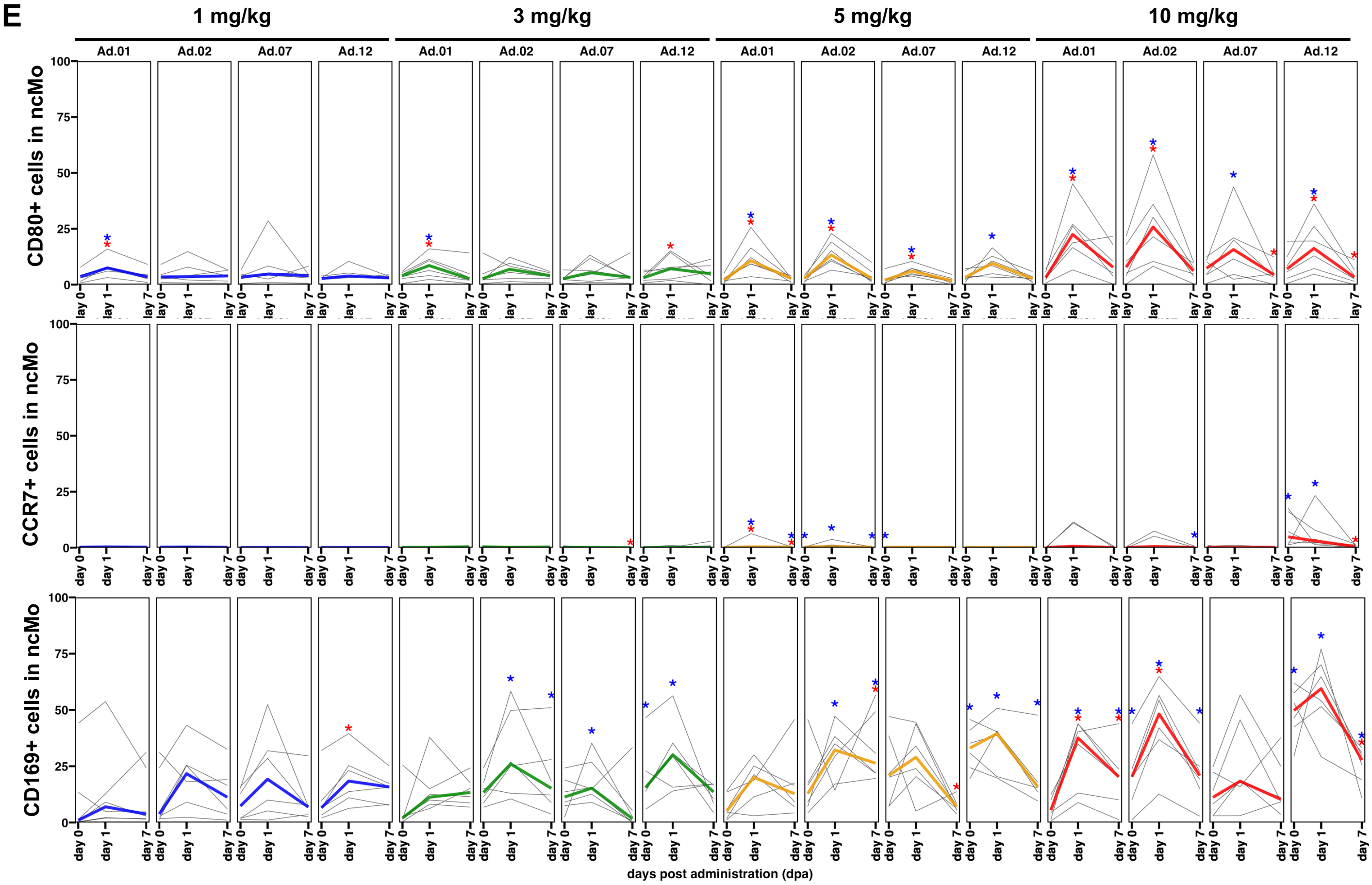

Supplemental Figure S7.

Dynamic profiling of monocyte subsets and activation status following repeated administration of SA-5 in cynomolgus macaques

A) Pie charts showing the distribution of classical (cMo), intermediate (iMo), and non-classical (ncMo) monocytes in peripheral blood at day 0 (d0), day 1 (d1), and day 7 (d7) after each administration (Ad.01, Ad.02, Ad.07, Ad.12) at the indicated doses.

B) Line plot indicates the percentage of cMo, iMo and ncMo among total monocytes at the same time points.

C) Line plots indicates the percentage of $CD80^{+}$, $CCR7^{+}$, and $CD169^{+}$ cells within the cMo subset across the treatment schedule.

D) Line plots indicates the percentage of $CD80^{+}$, $CCR7^{+}$, and $CD169^{+}$ cells within the iMo subset across the treatment schedule.

E) Line plots indicates the percentage of $CD80^{+}$, $CCR7^{+}$, and $CD169^{+}$ cells within the ncMo subset across the treatment schedule.

In all panels, bold lines represent group medians, and thin lines represent individual macaques. Colors indicate SA-5 dose groups (1, 3, 5, and 10 mg/kg).

(B-E) In all panels, bold lines represent group medians, and thin lines represent individual macaques. Colors indicate SA-5 dose groups (1, 3, 5, and 10 mg/kg). Statistical significance was determined using the paired Mann–Whitney *U* test for comparisons with day 0. Blue asterisks indicate comparisons with day 0 of Administration 1 (Ad.01), and red asterisks indicate comparisons with day 0 of each corresponding administration. (**p* < 0.05 , ***p* < 0.01).

****Abbreviations**:** cMo, classical monocyte; iMo, intermediate monocyte; ncMo, non-classical monocyte; CD, cluster of differentiation; Ad., administration; d0/d1/d7, day 0/day 1/day 7.

Supplemental Figure S8

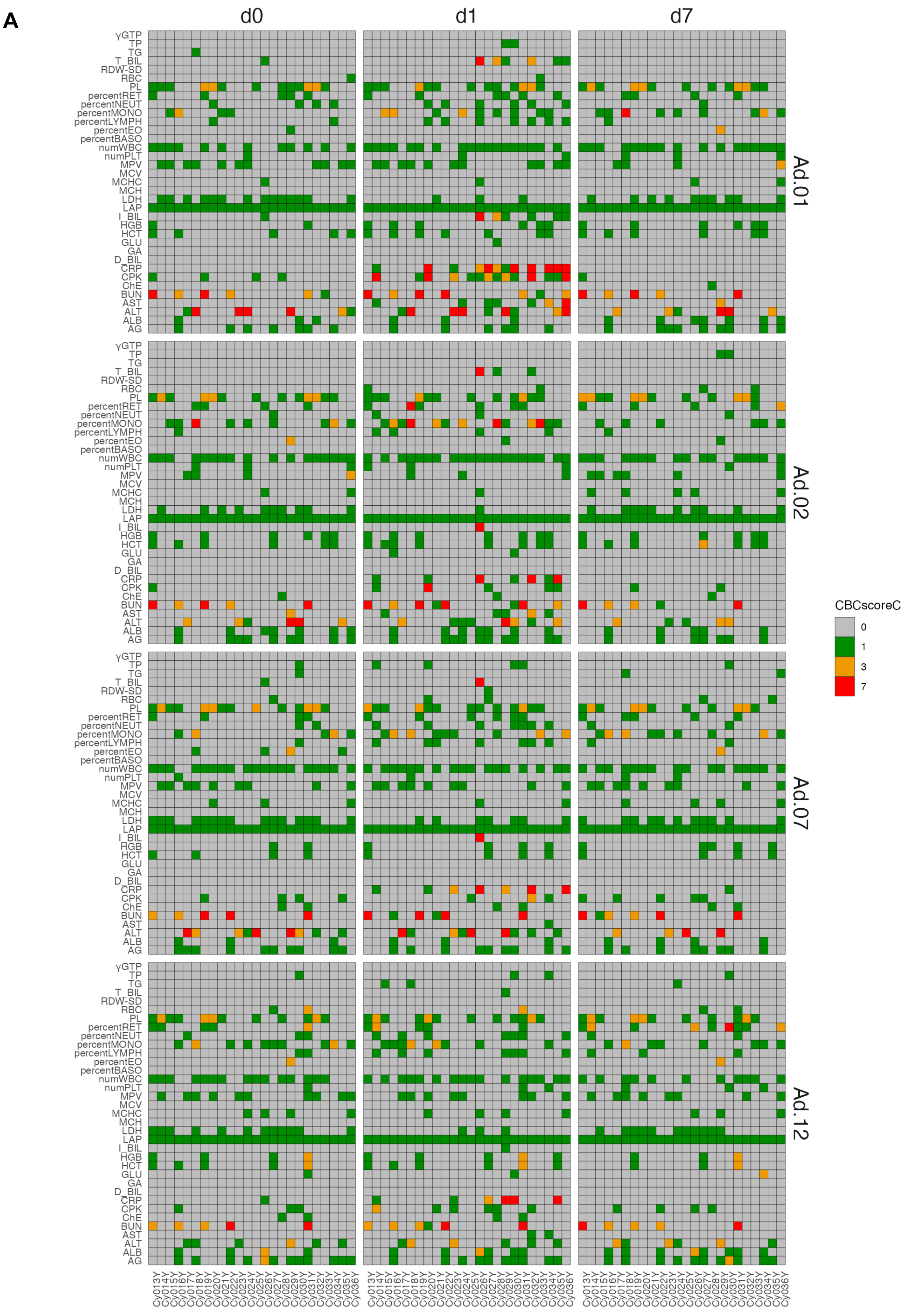

Supplemental Figure S8

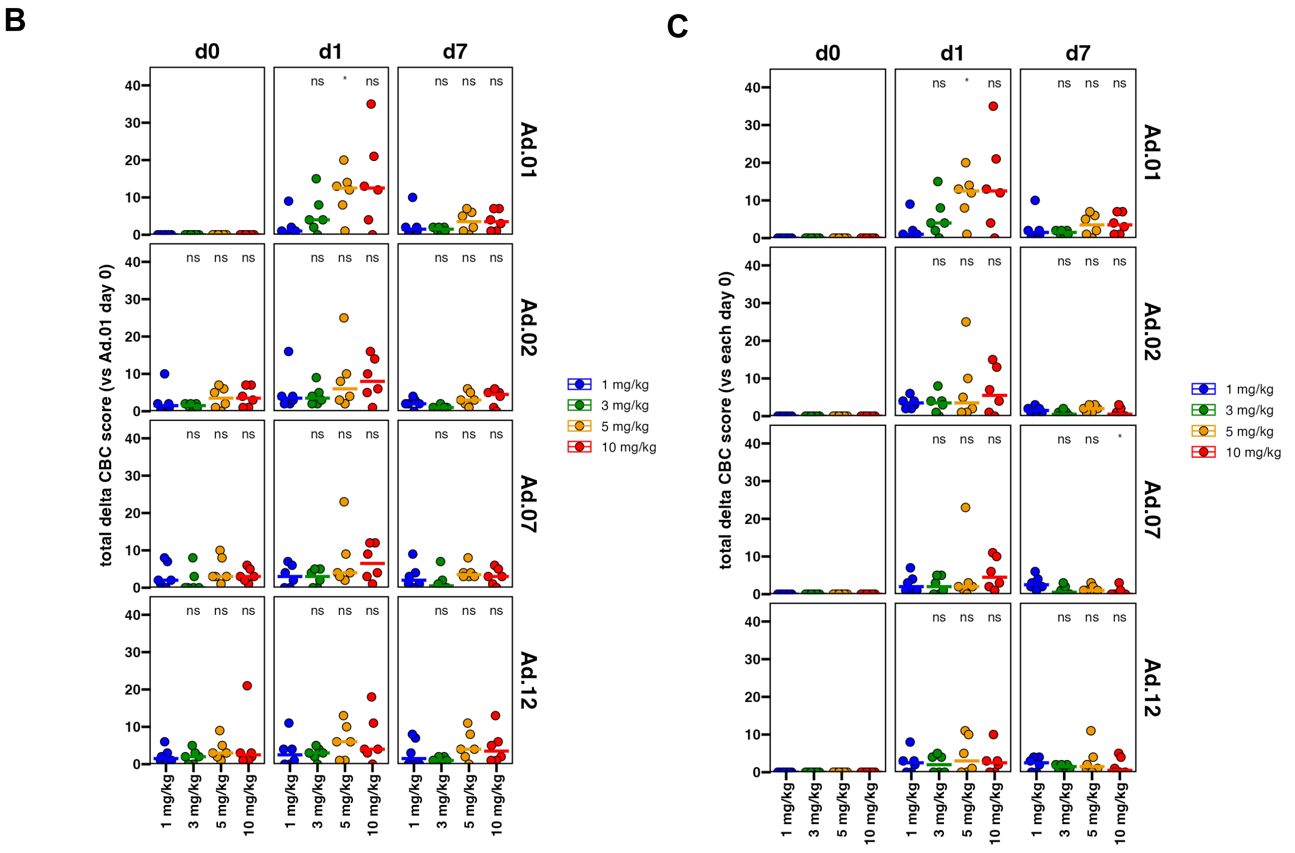

Supplemental Figure S8.
Individual and group-level deviation scores for hematological and clinical chemistry parameters following repeated administration of SA-5 in cynomolgus macaques
A) Heatmap showing individual deviation scores across 35 hematological and biochemical parameters at day 0 (d0), day 1 (d1), and day 7 (d7) following the 1st, 2nd, 7th, and 12th administrations (Ad.01–Ad.12). Deviation scores were assigned based on distance from reference ranges (score 0–7).
B) Dot plotsong total deviation scores in each animal at each time point (d0, d1, d7), calculated relative to Ad.01 day 0.
C) Dot plots showing total deviation scores relative to each administration's own baseline (same-day d0).
Each dot represents one macaque; horizontal bars indicate group medians. These analyses summarize the extent and dynamics of off-target effects throughout the treatment schedule.

(B,C) Statistical significance was determined using the Mann–Whitney *U* test for comparisons with the 1 mg/kg group at each corresponding time point (**p* < 0.05).

Abbreviations: d0/d1/d7, day 0/day 1/day 7; Ad., administration.

Supplemental Figure S9

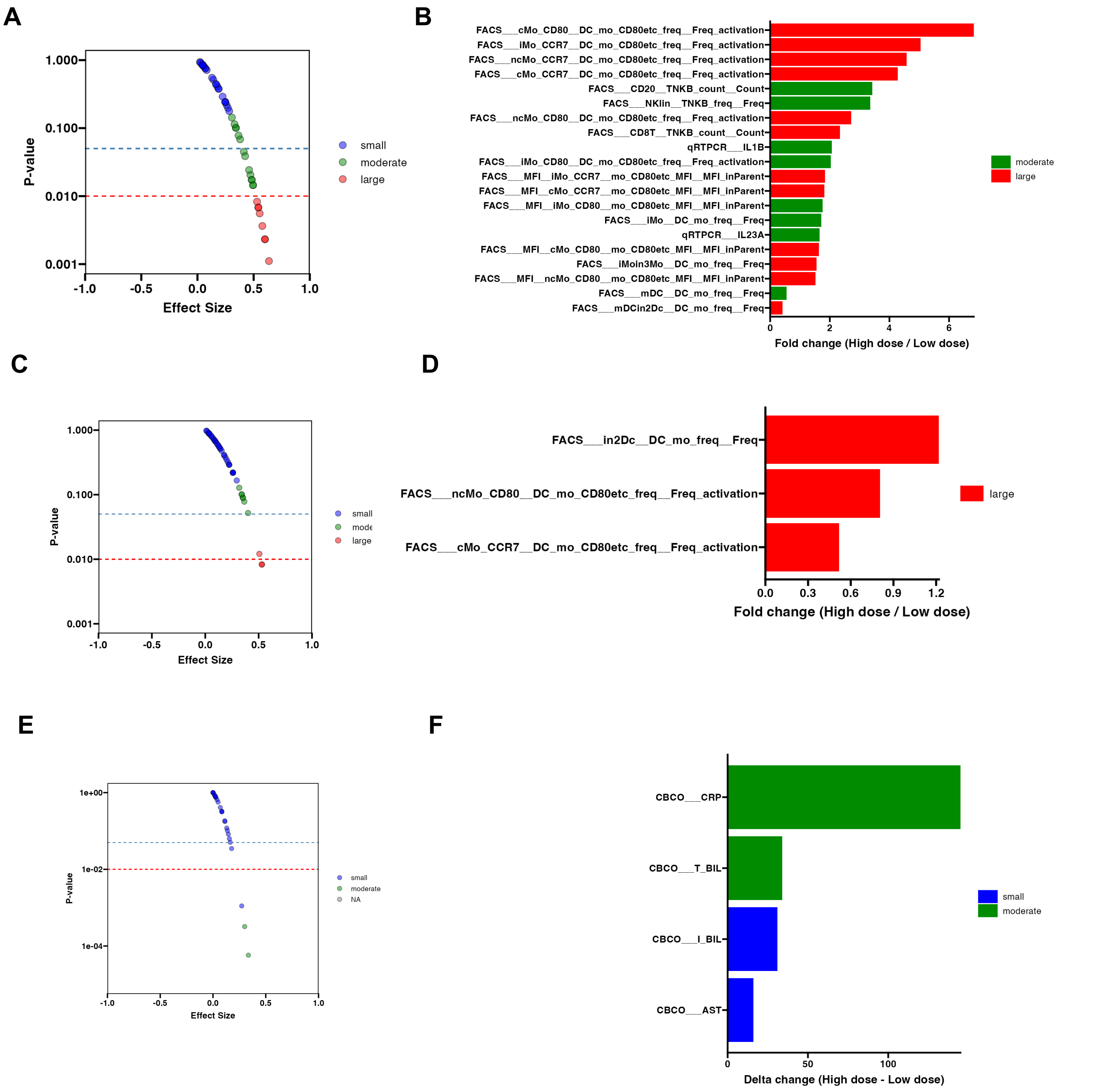

Supplemental Figure S9.
Effect size-based comparison of immune and clinical parameters between high- and low-dose SA-5 groups in cynomolgus macaques
A, C, E) Volcano-style plots displaying effect sizes (x-axis) and p-values (y-axis) for selected immune (A, C) and clinical (E) parameters. Points are color-coded by effect size magnitude: small (blue), moderate (green), and large (red).
B, D, F) Corresponding bar plots showing fold changes (B, D) or delta changes (F) between high-dose and low-dose groups.
B) Immune activation markers measured by flow cytometry.
D) Subset-specific activation markers in monocytes and dendritic cells.
F) Clinical biochemical parameters, including C-reactive protein (CRP), bilirubin, and aspartate aminotransferase (AST).
Colors indicate effect size classifications as in panels A, C, and E.

(A,C,E) Statistical significance was determined using the Mann–Whitney *U* test for comparisons detail described in methods.

****Abbreviations**:** AST, aspartate aminotransferase; CRP, C-reactive protein; FACS, fluorescence-activated cell sorting.

Supplemental Figure S10

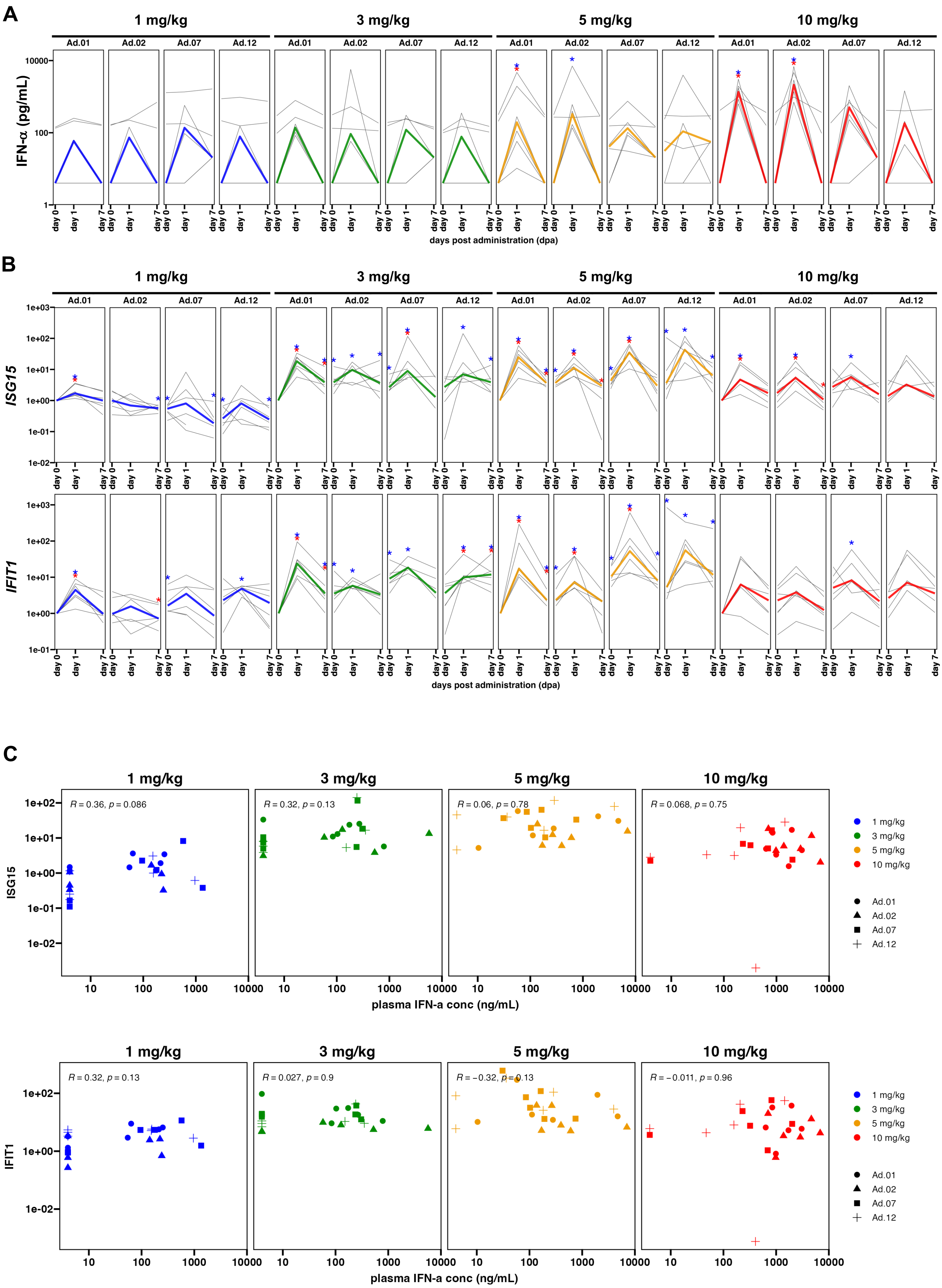

Supplemental Figure S10

D

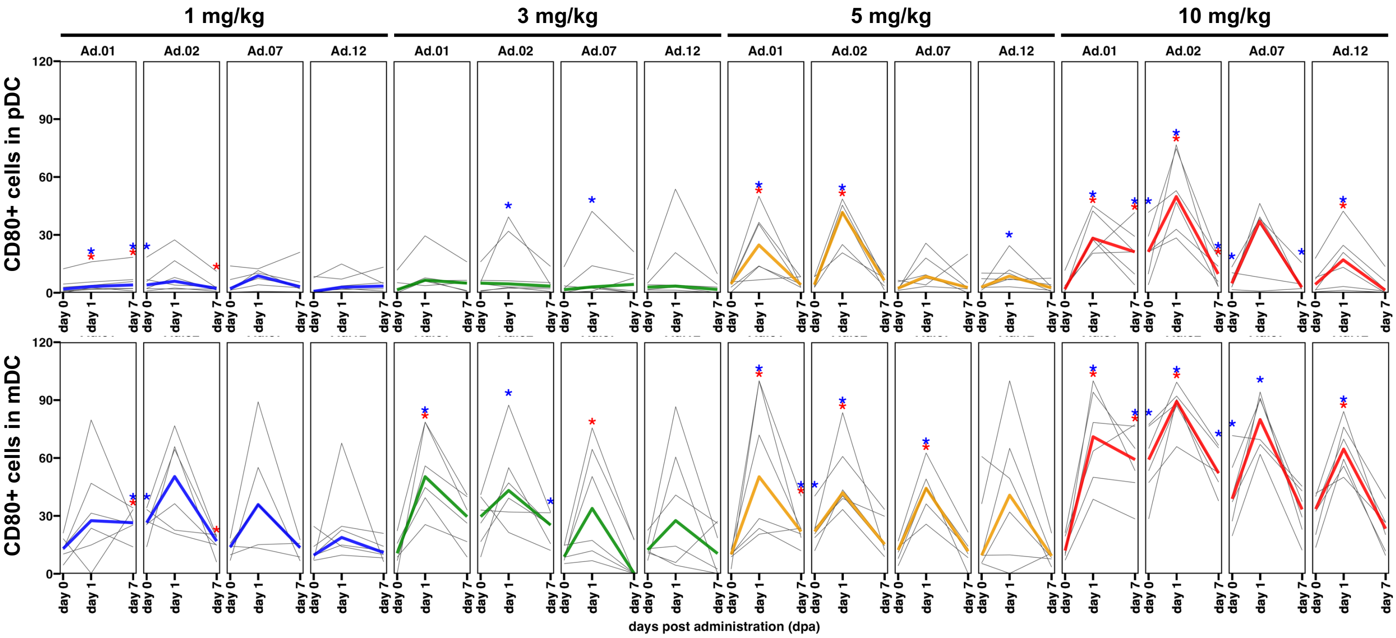

E

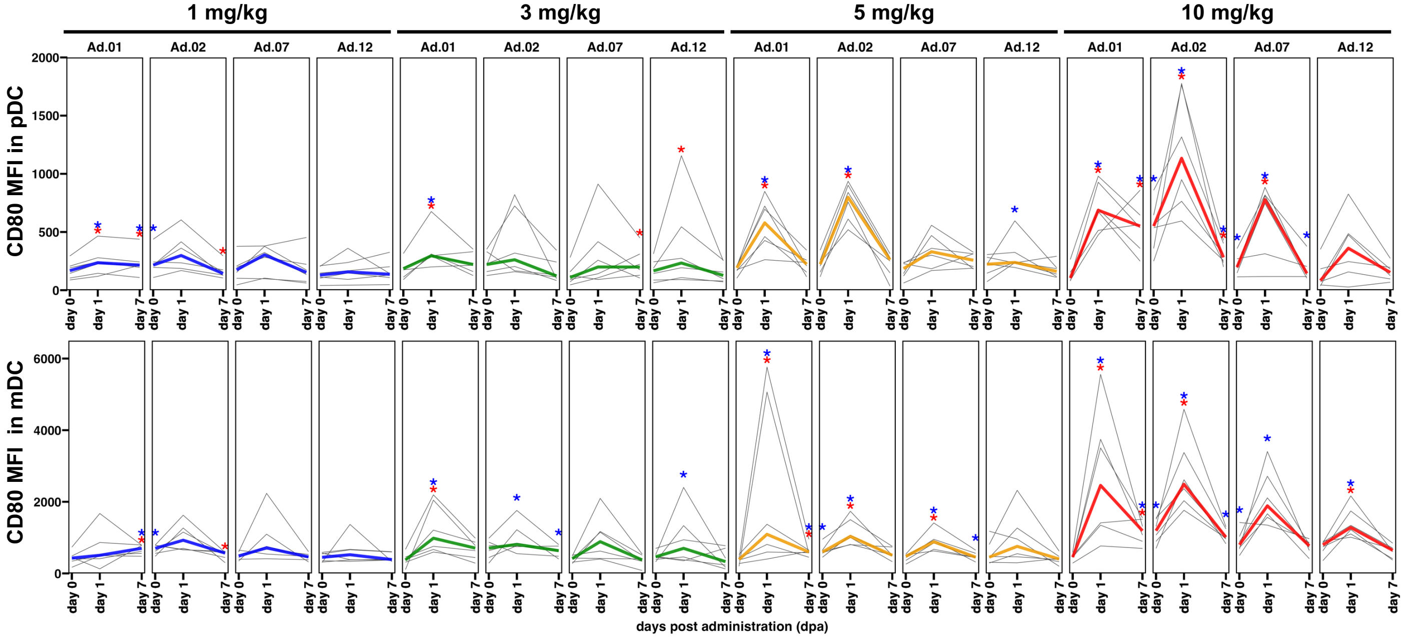

Supplemental Figure S10.

Integrated assessment of type I IFN signaling and dendritic cell activation following repeated administration of SA-5 in cynomolgus macaques

A) Line plots show plasma IFN-α concentrations measured by ELISA at day 0 (d0), day 1 (d1), and day 7 (d7) after each administration (Ad.01 to Ad.12). Values are plotted on a logarithmic scale.

B) Line plots show relative mRNA expression levels of ISG15 (top) and IFIT1 (bottom) in peripheral blood mononuclear cells (PBMCs) at the same time points, measured by quantitative reverse transcription PCR (qRT-PCR). Values are plotted on a logarithmic scale.

C) Scatter plots showing the correlation between plasma IFN-α levels and expression of ISG15 (top) or IFIT1 (bottom) on day 1. Each panel represents a different dose group. Spearman's correlation coefficients (R) and p-values are indicated.

D) Line plots show the percentage of $CD80^+$ cells among plasmacytoid dendritic cells (pDCs; top) and myeloid dendritic cells (mDCs; bottom) at each time point.

E) Line plots show CD80 median fluorescence intensity (MFI) in pDCs (top) and mDCs (bottom), indicating activation status across time points and dose groups.

In all panels, bold lines represent group medians, and thin lines represent individual macaques. Colors indicate SA-5 dose groups (1, 3, 5, and 10 mg/kg).

(A, B, D, E) In all panels, bold lines represent group medians, and thin lines represent individual macaques. Colors indicate SA-5 dose groups (1, 3, 5, and 10 mg/kg). Statistical significance was determined using the paired Mann–Whitney *U* test for comparisons with day 0. Blue asterisks indicate comparisons with day 0 of Administration 1 (Ad.01), and red asterisks indicate comparisons with day 0 of each corresponding administration. (*$p < 0.05$, **$p < 0.01$). (C) Statistical significance was determined using the Spearman's rank correlation test.

****Abbreviations**:** IFN, interferon; IFN-α, interferon-alpha; ISG15, interferon-stimulated gene 15; IFIT1, interferon-induced protein with tetratricopeptide repeats 1; PBMC, peripheral blood mononuclear cell; qRT-PCR, quantitative reverse transcription polymerase chain reaction; pDC, plasmacytoid dendritic cell; mDC, myeloid dendritic cell; MFI, median fluorescence intensity; ELISA, enzyme-linked immunosorbent assay; Ad., administration; d0/d1/d7, day 0/day 1/day 7.

Supplemental Figure S11

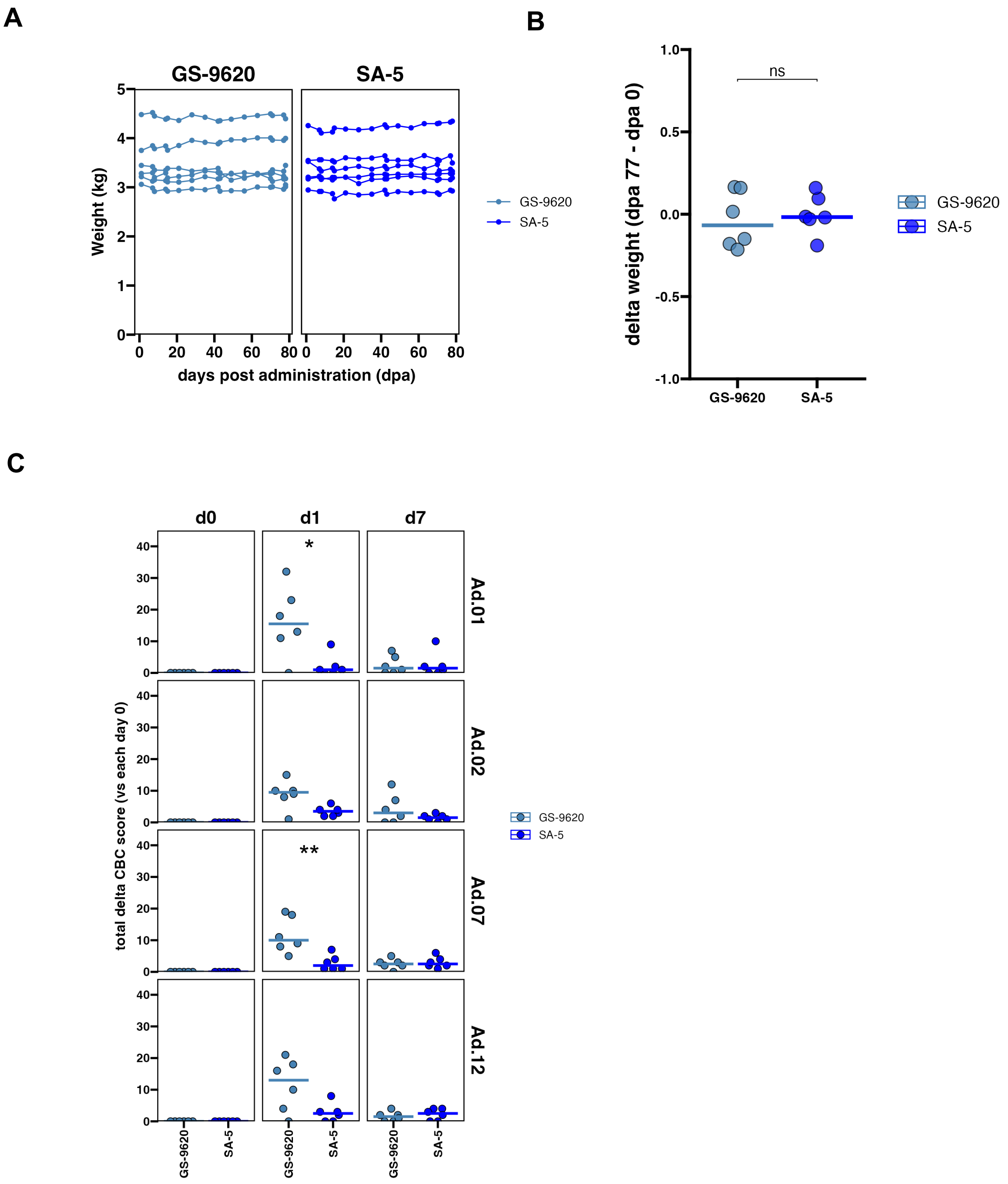

Supplemental Figure S11.
Comparison of safety-related parameters between SA-5 and GS-9620 in cynomolgus macaques
A) Line plots indicate changes in body weight during repeated administration of SA-5 and GS-9620. Each line represents an individual animal; bold lines indicate group medians.
B) Dot plot showing delta body weight (day 77 minus day 0) in each group. No statistically significant differences (the nonparametric Wilcoxon/Mann–Whitney *U* test) were observed between groups (ns).
C) Total deviation scores in hematological parameters at day 0 (d0), day 1 (d1), and day 7 (d7) after the 1st, 2nd, 7th, and 12th administrations (Ad.01 to Ad.12), compared between GS-9620 and SA-5. Deviation scores were calculated based on distance from reference ranges across 35 hematological parameters. Each dot represents an individual animal; horizontal bars indicate group medians.

(B, C) Statistical significance was determined using the Mann–Whitney *U* test for comparisons between GS-9620 and SA-5 (*p < 0.05, **p < 0.01).

Abbreviations: d0/d1/d7, day 0/day 1/day 7; Ad., administration; ns, not significant.

Supplemental Figure S12

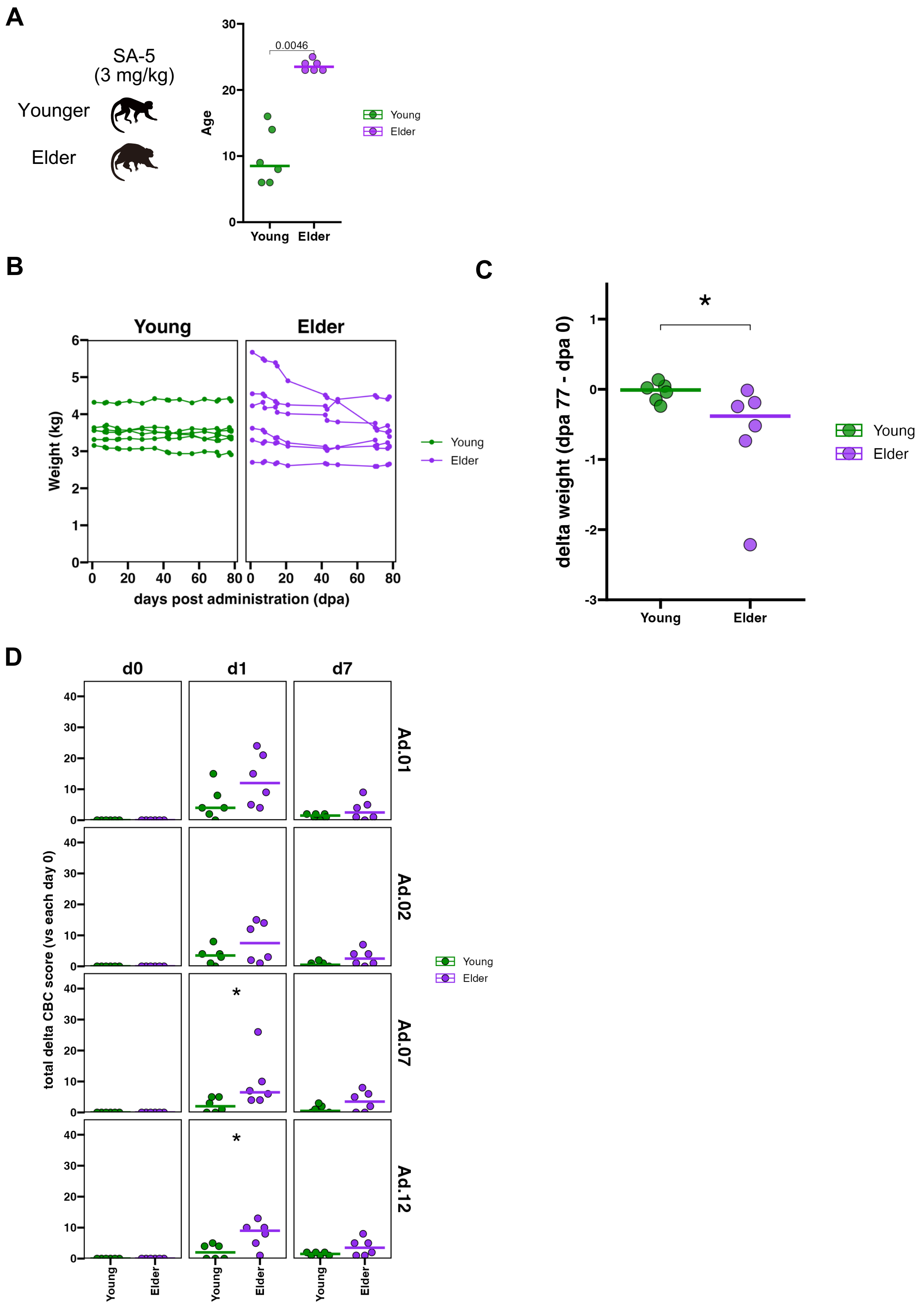

Supplemental Figure S12.
Comparison of safety-related parameters between young and aged cynomolgus macaques following administration of SA-5 (3 mg/kg)
A) Dot plot showing the age distribution of animals categorized as "young" or "elderly." A statistically significant difference in age was confirmed between the two groups (the nonparametric Wilcoxon/Mann–Whitney *U* test).
B) Line plots indicate changes in body weight throughout the treatment period in young and aged animals. Each line represents an individual animal; bold lines represent group medians.
C) Dot plot showing delta body weight (day 77 minus day 0) for each group. A statistically significant difference (the nonparametric Wilcoxon/Mann–Whitney *U* test) was observed between age groups (*p < 0.05).
D) Total deviation scores in hematological parameters at day 0 (d0), day 1 (d1), and day 7 (d7) after the 1st, 2nd, 7th, and 12th administrations (Ad.01 to Ad.12). Scores were calculated based on deviations from reference ranges across 35 clinical blood parameters. Each dot represents an individual macaque; horizontal bars indicate group medians.
(B, C) Statistical significance was determined using the Mann–Whitney *U* test for comparisons between Young and Elder (*p < 0.05, **p < 0.01).
Abbreviations: d0/d1/d7, day 0/day 1/day 7; Ad., administration.

Supplemental Table S1

Summary statistics for SA-5 pharmacokinetic parameters (cohort 1)

Toxicokinetics — Period : Treatment day 1
Sex : Male

			Plasma concentration of SA-5 (ng/mL)										
			Time (h)								C_{max}	t_{max}	$AUC_{0\text{-}t}$
Test article	Dose	Animal No.	1	2	4	6	8	24	48	72	(ng/mL)	(h)	(ng・h/mL)
SA-5	30 mg/kg	SP1M01	3.08	1.13	2.54	4.74	4.31	0.466	0.0402	0.0217	4.74	6.0	56.0
	100 mg/kg	SP1M02	1.98	1.02	0.799	1.37	4.42	1.32	0.131	<LLOQ	4.42	8.0	65.6
	300 mg/kg	SP2M01	31.3	10.1	6.26	4.21	4.26	1.42	1.82	0.409	31.3	1.0	172
	1000 mg/kg	SP2M02	7.15	7.64	15.3	7.32	30.0	6.07	3.10	1.34	30.0	8.0	489

LLOQ : The lower limit of quantification, 0.0200 ng/mL
<LLOQ: Below LLOQ

Toxicokinetics — Period : Treatment day 1
Sex : Female

			Plasma concentration of SA-5 (ng/mL)										
			Time (h)								C_{max}	t_{max}	$AUC_{0\text{-}t}$
Test article	Dose	Animal No.	1	2	4	6	8	24	48	72	(ng/mL)	(h)	(ng・h/mL)
SA-5	30 mg/kg	SP1F01	3.71	4.83	2.85	24.6	11.2	1.20	0.616	0.113	24.6	6.0	175
	100 mg/kg	SP1F02	6.06	4.57	7.29	5.64	18.9	2.44	1.85	0.122	18.9	8.0	253
	300 mg/kg	SP2F01	0.433	6.91	28.6	9.69	17.5	9.02	0.986	0.593	28.6	4.0	412
	1000 mg/kg	SP2F02	94.2	99.3	210	191	696	9.71	1.27	0.849	696	8.0	4440

LLOQ : The lower limit of quantification, 0.0200 ng/mL

Supplemental Table S1.
Summary statistics for SA-5 pharmacokinetic parameters in cynomolgus macaques
This table presents plasma concentrations and pharmacokinetic parameters of SA-5 following oral administration in male and female cynomolgus macaques. Plasma concentrations (ng/mL) were measured at multiple time points (1, 2, 4, 6, 8, 24, 48, 72 h) on day 1 of dosing. Pharmacokinetic metrics such as **Cmax** (maximum plasma concentration), **Tmax** (time to reach Cmax), and **AUC0–t** (area under the curve up to 48 hours) were calculated for each animal.
LLOQ: Lower limit of quantification = 0.0200 ng/mL.
< LLOQ: Below detection limit.

Supplemental Table S2

Summary statistics for SA-5 pharmacokinetic parameters (cohort 2)

Toxicokinetics Period : Treatment day 1
Sex : Male

Test article	Dose	Animal No.	Plasma concentration of SA-5 (ng/mL) Time (h) 2	4	8	24	48	C_{max} (ng/mL)	t_{max} (h)	AUC_{0-t} (ng・h/mL)
SA-5	10 mg/kg	TP2M01	1.08	0.392	4.72	0.605	0.0486	4.72	8	50.0
		TP2M02	4.53	2.09	2.11	1.43	0.881	4.53	2	74.4
		Mean	2.81	1.24	3.42	1.02	0.465	4.63	5.0	62.2
		n	2	2	2	2	2	2	2	2
	100 mg/kg	TP3M01	7.38	7.22	18.6	1.76	1.30	18.6	8	224
		TP3M02	3.59	3.30	3.03	1.61	1.36	3.59	2	94.6
		Mean	5.49	5.26	10.8	1.69	1.33	11.1	5.0	159
		n	2	2	2	2	2	2	2	2
	1000 mg/kg	TP4M01	29.3	51.2	16.9	6.45	1.96	51.2	4	498
		TP4M02	3.25	5.34	6.38	4.41	2.63	6.38	8	203
		Mean	16.3	28.3	11.6	5.43	2.30	28.8	6.0	351
		n	2	2	2	2	2	2	2	2

LLOQ : The lower limit of quantification, 0.0200 ng/mL

Toxicokinetics Period : Treatment day 29
Sex : Male

Test article	Dose	Animal No.	Plasma concentration of SA-5 (ng/mL) Time (h) 2	4	8	24	48	C_{max} (ng/mL)	t_{max} (h)	AUC_{0-t} (ng・h/mL)
SA-5	10 mg/kg	TP2M01	0.564	0.189	0.664	0.634	0.106	0.664	8	20.5
		TP2M02	2.16	1.22	0.921	0.405	0.255	2.16	2	27.8
		Mean	1.36	0.705	0.793	0.520	0.181	1.41	5.0	24.2
		n	2	2	2	2	2	2	2	2
	100 mg/kg	TP3M01	4.41	3.86	4.54	1.46	2.23	4.54	8	119
		TP3M02	7.61	4.78	1.27	2.88	0.725	7.61	2	102
		Mean	6.01	4.32	2.91	2.17	1.48	6.08	5.0	111
		n	2	2	2	2	2	2	2	2
	1000 mg/kg	TP4M01	39.7	58.7	40.4	2.58	1.72	58.7	4	607
		TP4M02	257	80.9	14.0	5.08	3.84	257	2	965
		Mean	148	69.8	27.2	3.83	2.78	158	3.0	786
		n	2	2	2	2	2	2	2	2

LLOQ : The lower limit of quantification, 0.0200 ng/mL

Toxicokinetics Period : Treatment day 1
Sex : Female

Test article	Dose	Animal No.	Plasma concentration of SA-5 (ng/mL) Time (h) 2	4	8	24	48	C_{max} (ng/mL)	t_{max} (h)	AUC_{0-t} (ng・h/mL)
SA-5	10 mg/kg	TP2F01	0.422	0.757	2.96	1.33	0.165	2.96	8	55.0
		TP2F02	12.4	2.90	13.6	1.03	0.0635	13.6	8	145
		Mean	6.41	1.83	8.28	1.18	0.114	8.28	8.0	100
		n	2	2	2	2	2	2	2	2
	100 mg/kg	TP3F01	0.464	0.469	1.13	0.161	0.452	1.13	8	19.9
		TP3F02	6.77	12.3	17.1	2.15	0.354	17.1	8	224
		Mean	3.62	6.38	9.12	1.16	0.403	9.12	8.0	122
		n	2	2	2	2	2	2	2	2
	1000 mg/kg	TP4F01	20.4	4.10	4.50	7.48	2.25	20.4	2	258
		TP4F02	88.6	452	61.0	2.93	0.858	452	4	1760
		Mean	54.5	228	32.8	5.21	1.55	236	3.0	1010
		n	2	2	2	2	2	2	2	2

LLOQ : The lower limit of quantification, 0.0200 ng/mL

Toxicokinetics Period : Treatment day 29
Sex : Female

Test article	Dose	Animal No.	Plasma concentration of SA-5 (ng/mL) Time (h) 2	4	8	24	48	C_{max} (ng/mL)	t_{max} (h)	AUC_{0-t} (ng・h/mL)
SA-5	10 mg/kg	TP2F01	0.188	0.203	0.994	0.410	0.0912	0.994	8	18.7
		TP2F02	1.66	0.572	0.618	0.616	0.314	1.66	2	27.0
		Mean	0.924	0.388	0.806	0.513	0.203	1.33	5.0	22.9
		n	2	2	2	2	2	2	2	2
	100 mg/kg	TP3F01	9.11	3.18	3.95	0.779	0.488	9.11	2	81.3
		TP3F02	4.98	3.51	1.87	4.18	0.350	4.98	2	110
		Mean	7.05	3.35	2.91	2.48	0.419	7.05	2.0	95.7
		n	2	2	2	2	2	2	2	2
	1000 mg/kg	TP4F01	22.8	18.4	7.00	8.66	4.96	22.8	2	401
		TP4F02	11.9	13.5	48.5	6.51	2.29	48.5	8	595
		Mean	17.4	16.0	27.8	7.59	3.63	35.7	5.0	498
		n	2	2	2	2	2	2	2	2

LLOQ : The lower limit of quantification, 0.0200 ng/mL

Supplemental Table S2

Supplemental Table S2.
Summary statistics for SA-5 pharmacokinetic parameters in cynomolgus macaques
This table presents plasma concentrations and pharmacokinetic parameters of SA-5 following oral administration in male and female cynomolgus macaques. Plasma concentrations (ng/mL) were measured at multiple time points (2, 4, 8, 24, 48 h) on day 1 or day 29 of dosing. Pharmacokinetic metrics such as **Cmax** (maximum plasma concentration), **Tmax** (time to reach Cmax), and **AUC0–t** (area under the curve up to 48 hours) were calculated for each animal.
LLOQ: Lower limit of quantification = 0.0200 ng/mL.
< LLOQ: Below detection limit.

Supplemental Table S3

Demographic and treatment information of macaques included in the GS9620 and SA5 adjuvant study

Monkey ID	Adjuvant	AdjuvantConc	AgeGroup	SEX	Age
Cy001Y	GS9620	0.5	Young	F	5
Cy002Y	GS9620	0.5	Young	F	6
Cy003Y	GS9620	0.5	Young	F	8
Cy004Y	GS9620	0.5	Young	M	13
Cy005Y	GS9620	0.5	Young	F	14
Cy006Y	GS9620	0.5	Young	F	16
Cy007Y	GS9620	1	Young	F	5
Cy008Y	GS9620	1	Young	F	6
Cy009Y	GS9620	1	Young	F	8
Cy010Y	GS9620	1	Young	M	13
Cy011Y	GS9620	1	Young	F	14
Cy012Y	GS9620	1	Young	F	16
Cy013Y	SA5	1	Young	M	6
Cy014Y	SA5	1	Young	M	6
Cy015Y	SA5	1	Young	M	8
Cy016Y	SA5	1	Young	M	9
Cy017Y	SA5	1	Young	F	14
Cy018Y	SA5	1	Young	F	16
Cy019Y	SA5	3	Young	M	6
Cy020Y	SA5	3	Young	M	6
Cy021Y	SA5	3	Young	M	8
Cy022Y	SA5	3	Young	M	9
Cy023Y	SA5	3	Young	F	14
Cy024Y	SA5	3	Young	F	16
Cy025Y	SA5	5	Young	F	5
Cy026Y	SA5	5	Young	F	6
Cy027Y	SA5	5	Young	F	8
Cy028Y	SA5	5	Young	M	13
Cy029Y	SA5	5	Young	F	14
Cy030Y	SA5	5	Young	F	16
Cy031Y	SA5	10	Young	M	6
Cy032Y	SA5	10	Young	M	6
Cy033Y	SA5	10	Young	M	8
Cy034Y	SA5	10	Young	M	9
Cy035Y	SA5	10	Young	F	14
Cy036Y	SA5	10	Young	F	16
Cy037E	SA5	3	Elder	F	23
Cy038E	SA5	3	Elder	F	23
Cy039E	SA5	3	Elder	M	24
Cy040E	SA5	3	Elder	F	25
Cy041E	SA5	3	Elder	F	23
Cy042E	SA5	3	Elder	F	24

Supplemental Table S3

Supplemental Table S3.
Demographic and treatment information of cynomolgus macaques included in the GS-9620 and SA-5 study
This table provides individual-level data for cynomolgus macaques included in the immunomodulatory compound comparison study using GS-9620 and SA-5. For each animal, the following information is provided:
- **Monkey ID**
- **Compound used** (GS9620 or SA-5)
- **Compound concentration (mg/kg)**
- **Age group** (young or elder)
- **Sex**
- **Chronological age (in years)**

This dataset was used to stratify experimental groups and assess age-related or dose-dependent differences in safety and immunological responses.

Supplemental Table S4

Flow cytometry panel for surface marker profiling of immune cell subsets.

Antigen	Clone	Fluorochrome	Company	Cat#	RRID	Staining
**CD169 (Siglec-1)**	7-239	BUV395	BD	742997	AB_2741195	Surface
**CD8 (CD8a)**	RPA-T8	BUV563	BD	612914	AB_2870199	Surface
**HLA-DR**	G46-6	BUV661	BD	565073	AB_2722500	Surface
**CD83**	HB15e	BUV737	BD	564441	AB_2738809	Surface
**CD14**	M5E2	BUV805	BD	565779	AB_2716868	Surface
**CD66abce**	TET2	VioBlue	Miltenyi	130-119-851	AB_2751888	Surface
**CD16**	3G8	BV510	BioLegend	302048	AB_2562085	Surface
**CD282 (TLR2)**	11G7	BV605	BD	742768	AB_2741032	Surface
**CD25 (IL2RA)**	BC96	BV650	BioLegend	302634	AB_2563807	Surface
**CD20**	2H7	BV711	BioLegend	302342	AB_2562602	Surface
**CD80**	L307.4	BV750	BD	747001	AB_2871776	Surface
**CD4**	L200	BV786	BD	563914	AB_2738485	Surface
**CD40**	5C3	Alexa488	BioLegend	334318	AB_1501188	Surface
**CD69**	FN50	BB700	BD	747520	AB_2744097	Surface
**CD197 (CCR7)**	G043H7	PE	Biolegend	353204	AB_10913813	37℃
**CD11c**	3.9	PE-CF594	BD	565920	AB_2869734	Surface
**CD95**	DX2	Cy5PE	BD	559773	AB_397317	Surface
**CD123 (IL3Ra)**	7G3	Cy7PE	BD	560826	AB_10563898	Surface
**CD159a (NKG2A)**	Z199	APC	Beckman	A60797	AB_10643105	Surface
**CD28**	CD28.2	Ax700	BioLegend	302920	AB_528786	Surface
**CD3**	SP34-2	Cy7APC	BD	557757	AB_396863	Surface

Supplemental Table S4.
Flow cytometry panel for surface marker profiling of immune cell subsets
This table summarizes the antibodies used for flow cytometry-based phenotypic profiling of immune cell subsets in cynomolgus macaque peripheral blood mononuclear cells (PBMCs). For each antibody, the antigen target, clone name, fluorochrome, manufacturer, catalog number (Cat#), RRID (Research Resource Identifier), and staining condition are listed.
All markers were stained on the cell surface unless otherwise indicated. CD197 (CCR7) was stained at 37° C.
This panel was used for the multiparameter analysis of immune activation, differentiation status, and subset identification in both baseline and post-dosing samples.
